# Isoforms of U1-70k Control Subunit Dynamics in the Human Spliceosomal U1 snRNP

**DOI:** 10.1371/journal.pone.0007202

**Published:** 2009-09-28

**Authors:** Helena Hernández, Olga V. Makarova, Evgeny M. Makarov, Nina Morgner, Yutaka Muto, Daniel Pomeranz Krummel, Carol V. Robinson

**Affiliations:** 1 Department of Chemistry, University of Cambridge, Cambridge, United Kingdom; 2 Department of Biochemistry, University of Leicester, Leicester, United Kingdom; 3 Division of Bioscience, School of Health and Social Care, Brunel University, Uxbridge, United Kingdom; 4 MRC Laboratory of Molecular Biology, Cambridge, United Kingdom; University Paris 7, France

## Abstract

Most human protein-encoding genes contain multiple exons that are spliced together, frequently in alternative arrangements, by the spliceosome. It is established that U1 snRNP is an essential component of the spliceosome, in human consisting of RNA and ten proteins, several of which are post-translationally modified and exist as multiple isoforms. Unresolved and challenging to investigate are the effects of these post translational modifications on the dynamics, interactions and stability of the particle. Using mass spectrometry we investigate the composition and dynamics of the native human U1 snRNP and compare native and recombinant complexes to isolate the effects of various subunits and isoforms on the overall stability. Our data reveal differential incorporation of four protein isoforms and dynamic interactions of subunits U1-A, U1-C and Sm-B/B'. Results also show that unstructured post-translationally modified C-terminal tails are responsible for the dynamics of Sm-B/B' and U1-C and that their interactions with the Sm core are controlled by binding to different U1-70k isoforms and their phosphorylation status *in vivo*. These results therefore provide the important functional link between proteomics and structure as well as insight into the dynamic quaternary structure of the native U1 snRNP important for its function.

## Introduction

The spliceosome catalyzes the removal of introns and the splicing together of exons from precursor-mRNA transcripts (pre-mRNA splicing). The human spliceosome, reported to contain over 140 subunits [1], undergoes extensive structural changes during its reaction cycle in which large complexes dissociate and others associate. Rearrangements of RNA are catalysed by helicases, and dynamic changes are mediated, possibly by varied post-translational modifications of the protein subunits [2]. These factors make this macromolecular ‘machine’ a challenging subject for biochemical and structural analysis.

Integral to the spliceosome are five U snRNPs (*u*ridine-rich *s*mall *n*uclear *r*ibo-*n*ucleo*p*roteins; U1, U2, U4, U5 and U6 snRNPs) and numerous non-snRNP associated splicing factors [3]. The U snRNPs carry out many essential functions via interactions of their RNA and protein components with the pre-mRNA; specifically, they mediate the recognition and subsequent pairing of the 5′ and 3′ splice sites of an intron. A critical initial step in pre-mRNA splicing involves recognition of the junction between the 5′ exon and intron (the 5′ splice site) by the U1 snRNP. This step acts to initiate formation of the spliceosome onto the pre-mRNA and represents a stage in the reaction that is highly subject to regulation (alternative splicing). Mammalian U1 snRNP consists of a 165-nucleotide RNA (U1 snRNA) and ten distinct proteins. U1 snRNA has an important functional role, its single-stranded 5′ end base-pairs to the 5′ splice site of the pre-mRNA. In addition, U1 snRNA serves a scaffold-like function for the binding of the ten protein subunits. Seven of the proteins called Sm proteins (Sm-E, F, G, B, D1, D2 and D3), are common to four of the U snRNPs while three proteins (U1-70k, U1-A and U1-C) are specific to U1 snRNP. Sm proteins recognize a short single-stranded region in U snRNAs (the Sm site). Their assembly at this site is critical to the biogenesis and assembly of U snRNPs [4]–[7] and *in vivo* their assembly onto the Sm site is promoted by the survival motor neuron protein (SMN) complex (reviewed in [8]. In neurons an additional Sm protein, termed Sm-N, has been identified but its tissue specific role is currently unclear [9]. Interestingly, in contrast to SmB which is expressed in all tissues examined to date, and SmB' which is widely expressed with the notable exception of the brain, SmN is found predominantly in central neurons.

Of the U1 snRNP specific proteins, U1-70k and U1-C have important roles in aiding recognition of the pre-mRNA transcript. U1-70k has an N-terminus that while highly conserved is predicted to be unstructured (residues ∼2–60), an RNA binding domain (or RBD) that mediates its interaction with a stem-loop of U1 snRNA (residues 92–202), and a C-terminus rich in repeats of arginine and serine residues (an RS ‘domain’) as well as R-(D/E) residues. Although this C-terminal domain is not conserved the RS ‘domain’ is important for interaction with non-snRNP splicing factors such as ASF/SF2 [10], [11]. Serines in this region are subject to post-translational modification (phosphorylation) and are important to splicing activity [12]. U1-C consists of an N-terminal zinc-finger domain and a C-terminal region rich in repeats of RG residues. Arginines in this region of U1-C are subject to post-translational modification (methylation) [13]. In contrast to U1-70k, U1-C does not bind to free U1 snRNA but requires the prior binding of the Sm proteins and U1-70k [14]. Mutations in the zinc-finger region of U1-C have a significant effect on recognition of the 5′ spice site by the U1 snRNP [15], indicating that this protein has a direct role to play in this activity.

Our understanding of the assembly and function of U1 snRNP has been greatly enhanced initially by cryo electron microscopy studies [16] and more recently by elucidation of its three-dimensional structure by X-ray crystallography [17]. Previously, crystal structures of four of seven Sm proteins led to the modeling of the remaining three (Sm-F, Sm-E and Sm-G) and the proposal that together they would interact to form a seven-membered ring [18]. The crystal structure of a completely recombinant human U1 snRNP revealed that Sm proteins do form a heptameric ring, composed of a single copy of each Sm protein, and passing through its center is the Sm site of U1 snRNA [17]. In the crystal structure U1-C is in a position to recognize the duplex formed when the 5′ end of U1 snRNA base-pairs to the 5′ splice site. The finding that the N-terminus of U1-70k extends 180 Å from the RBD and wraps around one face of the Sm ring, crossing Sm-D2 and Sm-D3/B, could therefore ensure the correct structure and positioning of U1-C for interaction with the U1 snRNA:5′ splice site duplex.

Many outstanding issues remain however such as the effects on dynamics, interactions, and stability of the particle when proteins such as U1-70k and U1-C undergo post-translational modification or when different isoforms of the U1 snRNP proteins are incorporated. It is established for example that two different splice variants of SmB are present (SmB/B') although their relative incorporation in the intact particle is not known [19], [20]. Given that the intact particle contains U1 snRNA and the finding that of the four different isoforms of U1-70k [21], [22] only two contain RNA binding domains (residues 92–202) this restricts the number of isoforms in the intact particle to two: U1-70k isoform 1 and U1-70k isoform 2 (supplementary text S1) [23]. Coincidentally the isoforms of U1-70k (1 and 2) and SmB/B' both differ by nine residues. U1-70k isoform 1 however includes a known phosphorylation site (Ser226). The effects of these isoforms, their phosphorylation status and the effects of other post translational modifications on the structure and dynamics of U1 snRNP are currently unknown.

To investigate this we have applied an emerging mass spectrometry approach which is becoming increasingly important for studying intact functional complexes [24]. By maintaining protein complexes intact and actively generating multiple sub-complexes with overlapping components, it is possible to determine the complete subunit architecture of a cellular complex. Using this approach, structural models of the yeast exosome [24], the yeast 19S proteasome lid [25], and the human elongation initiation factor 3 [26] have been proposed. Interestingly, the crystal structure of the human exosome, formed by expression and reconstitution of nine of its ten subunits [27], in comparison to mass spectrometry (MS) data for the native homologous yeast complex, shows that the stable sub-complexes generated in solution correspond to those that form the largest subunit interfaces [28]. Moreover, the relatively low surface area of interaction of the peripheral subunits was found to correlate with their propensity to dissociate. This MS approach, of generating sub-complexes to define subunit interaction maps and interface strengths, has to date been largely restricted to multi-protein complexes due to difficulties inherent in recording spectra of sufficient resolution for protein-RNA complexes [26], [29]. Further, isoforms and multiple post-translational modifications observed for several of the U1 snRNP proteins, present challenges to the study of this particle. Since the majority of the post-translational modifications are likely to be sub-stoichiometric, and given the presence of several isoforms, considerable heterogeneity in this cellular protein-RNA complex is anticipated.

Here we show that despite the heterogeneity of human U1 snRNP we can obtain well-resolved mass spectra of the intact complex. Using U1 snRNP isolated directly from HeLa cells, such that interactions between its snRNA and ten proteins are preserved, we compare this native complex with a completely recombinant one [17], [30] revealing the effects of various truncated proteins and native isoforms on the overall stability of the U1 snRNP. Our results show that complexes containing the larger of two U1-70k isoforms, U1-70k isoform 1 which has the additional phosphorylation site, are more prevalent *in vivo*. We also show that the two Sm-B isoforms (Sm-B/B'), are of equal abundance. Interestingly however interactions of U1-70k isoform 1 with U1-C are enhanced relative to those of isoform 2. Conversely, interactions of Sm-B/B' with U1-70k isoform 2 are enhanced relative to those of isoform 1.

## Results

### Mass spectra of U1 snRNP reveal its heterogeneity

**Figure 1:**
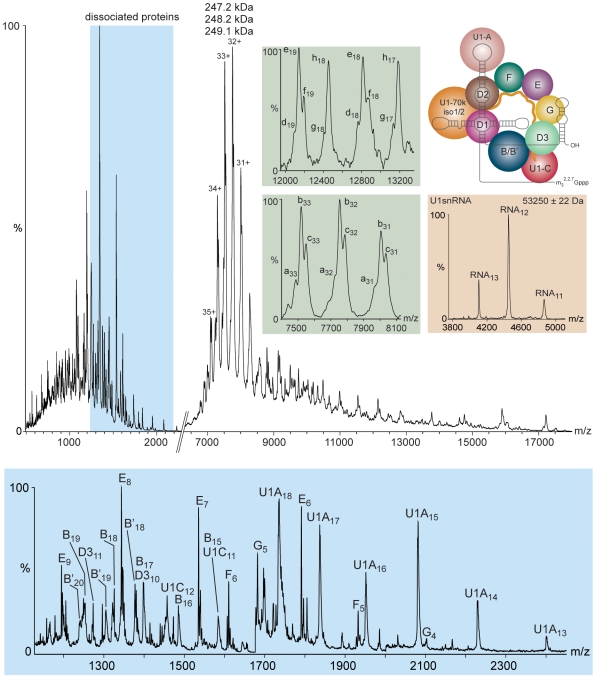
Nano ESI mass spectra of the cellular U1 snRNP showing intact U1 snRNP as well a series of dissociated individual proteins (blue) and the U1snRNA after release from the complex (beige). The charge states assigned to the intact complex correspond in mass to: 247.2, 248.2 and 249.1 kDa. An expansion of the charge states assigned to the intact complex reveals at least three different forms (a–c) (green). Dissociation of individual proteins from the intact complex gives rise to doublets (g–h) and triplets (d–f) in the peaks assigned to the sub-complex products (green). A schematic model of the U1 snRNP architecture is shown [17] where iso1/2 indicates U1-70k isoform 1 or 2 and B/B' refers to Sm-B or Sm-B'. MS (QToF2) conditions: capillary: 1.5 kV, cone: 200 V, extractor: 0 V, collision cell voltage: 100 V, source transfer region readback: 7.1×10^−3^ mbar, ToF readback: 1.3×10^−6^ mbar. Inset conditions as main spectrum except collision cell voltage 130 V.

To define the composition of native human U1 snRNP initially we carried out a proteomic analysis. All ten of the anticipated proteins were detected and additionally two from the U2 snRNP (Sm-A' and Sm-B″, see supplementary text S1). Subsequently, we determined the masses of the intact U1 proteins after chromatographic separation of the denatured complex and electrospray ionisation (ESI) MS. We repeated the separation process to determine the amino acid sequence of tryptic peptides enabling us to identify the Sm proteins and to correlate identity with intact masses [26], [31] (table S1). Three of the subunits (U1-70k, Sm-D1 and Sm-D2) were not observed using this approach. Phosphorylation of U1-70k and its removal with U1 snRNA has been suggested previously for the absence of U1-70k in preparations of cellular extracts [32]. However since U1-70k, Sm-D1 and Sm-D2 were readily identified in our proteomics analysis we used database values, considering the two U1-70k isoforms with RNA binding domains: U1-70k isoforms 1 and 2 (table S1) [21], [22]. The mass of the snRNA component of the U1 snRNP complex was determined after a phenol/chloroform extraction of the nucleic acid followed by an ethanol precipitation (supplementary text S1). Re-suspension in aqueous buffer and MS confirms the presence of one species with a mass determined experimentally (53250±22 Da) close to that predicted for the established sequence (53271 Da) [33] (figure 1 inset). Summation of the masses of the 10 protein subunit, seven determined empirically and three from databases, together with the lowest mass protein isoforms and the measured mass of the snRNA, leads to the lowest calculated mass for the intact complex as 245806 Da (table S2).

Having established the composition of the cellular U1 snRNP and defined the lowest anticipated mass we recorded nano ESI mass spectra of the intact complex (figure 1 and table S3). Despite the predicted presence of multiple sub-stoichiometric phosphorylation, methylation and metal binding sites together with the incorporation of the different isoforms and the presence of RNA, the mass spectrum of native human U1 snRNP is remarkably well resolved. The intact complex was observed over charge states 35+ to 31+ (figure 1); each charge state comprised a triplet of peaks corresponding in mass to 247.2, 248.2, and 249.1 kDa Activation in the gas phase was required to improve the resolution of the spectrum and under these conditions individual proteins dissociate. The dominant peaks for the individual proteins correspond to the Sm ring proteins Sm-E, Sm-F, Sm-G, Sm-B/B', Sm-D3, and the U1 snRNP specific proteins U1-C and U1-A. The ease of dissociation of individual proteins in the gas phase of the mass spectrometer is governed primarily by their propensity to unfold and accumulate charge [34]. As a consequence proteins that are buried [35], have a high molecular mass, interact with a large number of subunits [26], and/or are involved in electrostatic interactions with RNA [36] are less likely to unfold and dissociate. Interestingly, despite their relatively low mass, the subunits Sm-D1 and Sm-D2 do not dissociate under these conditions implying that they are protected by virtue of extensive interactions with each other, the snRNA, and/or U1-70k.

Given the established tendency for protein complexes to retain water/buffer molecules within subunit interfaces [37] this lowest calculated mass is in accord with the lowest mass determined experimentally (247167±56 Da; figure 1). The predominant U1 snRNP species observed in the spectra is therefore consistent with the presence of one copy of U1 snRNA and each of the 10 protein subunits present at unit stoichiometry in accord with previous predictions ([38] and tables S1-2). Moreover, the relative abundance of complexes with masses lower than 247 kDa rules out the possibility of extensive sub-stoichiometric binding of proteins in the cellular complex.

### Four different isoforms are present simultaneously

An expansion of the peaks assigned to the intact complex reveals that they consist of multiple components (a, b and c) close to the mass of the intact U1 snRNP but separated by average mass differences of a–b 1005±33 Da and b–c 922±28 Da (figure 1, inset green; table S3). Likely candidates for these mass differences are proteins having multiple post-translational modifications (PTMs) and/or existing in different isoforms. Considering first the possibility of multiple PTMs, four Sm proteins are reported to contain dimethylarginines; the masses determined in this study for three of these (Sm-B, Sm-B' and Sm-D3) indicate that they are present as single species i.e. ∼100% modified in agreement with previous reports; [39]–[41]. In the case of Sm-D1 the expected nine dimethylarginine residues [39], [41] give a maximum increase of 252 Da over the mass calculated from the amino acid sequence. At least six phosphorylation sites have been identified in U1-70k isolated from HeLa cells consistent with a mass increase of 480 Da ([42]–[46] and table S4). From the PTMs observed in this study and those reported in databases it is apparent that, if all modifications are present simultaneously, the maximum increment would be 732 Da. Since other post-translational modifications may be present but not yet reported this represents a conservative estimate of the mass increase.

**Figure 2:**
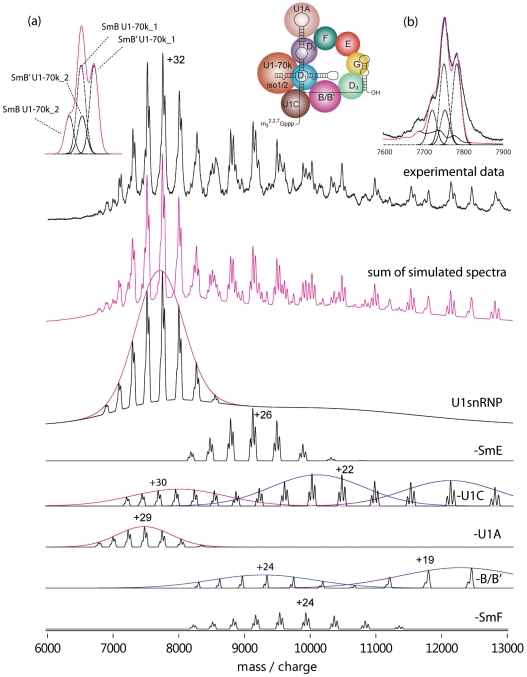
Simulation of the components of the spectrum recorded under higher activation conditions: (a) The convolution of the four isoforms U1-70k_1, U1-70k_2, Sm-B and Sm-B' gives rise to a triplet of peaks. The width of these peaks was selected based on the experimental peak width for the 32+ charge state: a similar profile is expected for all charge states. (b) Fitting of these Gaussian distributions to experimental data recorded for the subcomplexes and intact particle. Main panel: the charge state distributions of the various components fitted to Gaussian distributions of charge states leading to distinction between solution phase products (pink envelope, -U1C) and losses of partially and more unfolded subunits (blue and purple envelopes, -U1C and -B/B') respectively. Summation of the charge state distributions modelled for the intact complex and multiple sub-complexes generates a simulated spectrum (pink) in close agreement with the observed data (top spectrum, black).

Given the low probability that all sites will be fully phosphorylated simultaneously and since well-defined peak splitting is observed, rather than multiple partially modified forms, the most likely explanation is the presence of different isoforms. The mass differences between isoforms Sm-B and Sm-B' and U1-70k isoforms 1 and 2 are 1012 Da and 939 Da respectively (table S1). These values are very close to the mass difference between the triplet peaks observed for the charge states of the intact complex. However two sub-units each with two isoforms would be expected to generate four possible versions of the intact U1snRNP complex, with calculated masses ranging from 245806 Da to 247757 Da (table S2). This would lead to combinations of Sm-B'/U1-70k isoform 2 and Sm-B/U1-70k isoform 1 differing in mass by only 73 Da (246818 Da and 246745 Da). This mass difference corresponds to m/z differences of only 2.1–2.4 for the intact complex (charge states 35+ to 31+) (figure 1). This would therefore result in co-incident peaks from these two isoform combinations and consequently three peaks, rather than four would be observed (figure 2a), with m/z separations between the peaks equivalent to ∼1 kDa.

To determine the ratio of the isoforms in U1 snRNP we denatured the complex and used UV absorbance of the two Sm-B:Sm-B' isoforms separated chromatographically. A ratio of Sm-B:Sm-B' of 1∶1 was determined (figure S1). This equimolar ratio can be used to establish the relative abundance of the two U1-70k isoforms. The 1∶1 ratio of the Sm-B/B' isoforms established above enables us to deconvolute the U1-70k isoform 1: isoform 2 relative abundance ratio as 70∶30, with a standard deviation of ±1 (figure 2b, supplementary text S1, table S5).

We introduced the same solution of U1 snRNP, but at higher activation energy, to induce additional dissociation and consequently to increase the intensity of the peaks assigned to ‘stripped’ sub-complexes (d,e,f and g,h, figure 1 inset) formed by loss of highly charged subunits. If we examine the ratios of the peaks d, e and f, assigned on the basis of mass difference to loss of U1-C, we find the same pattern of splitting as peaks for the intact particle (a, b and c) the central peak being predominant consistent with sub-complexes containing the same ratio of different isoforms as the intact complexes (figure 1). By contrast for stripped complexes (g and h), formed by loss of Sm-B/B', the pattern is of only two peaks, the major one at higher m/z, as loss of Sm-B/B' isoforms reduces the multiplicity of the peaks.

The contribution of the various components to the overall mass spectrum was then assessed by simulating spectra for all products identified based on mass difference from the intact particle. Gaussian distributions for individual charge states were simulated (supplementary text S1 and table S5) taking into account contributions from water/buffer molecules to the overall peak widths. The extent of this contribution is adjusted according to the activation applied in the gas phase. Each charge state series is fitted to a Gaussian distribution to enable modelling of the relative intensities of the various components. The results of this simulation (figure 2) allow us to distinguish different charge state distributions and consequently to assign them to solution and gas phase dissociation products using the following assumption: sub-complexes that are formed in solution will appear in a similar m/z region of the spectrum to the intact complex, as a result of the charging that takes place in the electrospray droplet, which is related to their surface area [47]. We therefore deduce that both U1-A and U1-C dissociate readily in solution. By contrast dissociation of Sm-E and Sm-F gives rise to only one distribution of charge states, at higher m/z values than the intact complex. We conclude therefore that these two products are formed only in the gas phase, by the expulsion of highly charged Sm-E and Sm-F. Interestingly, two distributions are observed for losses of Sm-B/B' and U1-C in the gas phase, attributed to expulsion of partially unfolded and more extended conformers of U1-C and Sm-B/B'. Overall therefore simulation allow us to define dissociation of U1-A and U1-C in solution, and losses of U1-C and Sm-B/B' in the gas phase, in multiple conformations.

### Sub-complexes reveal the strength of interactions

**Figure 3:**
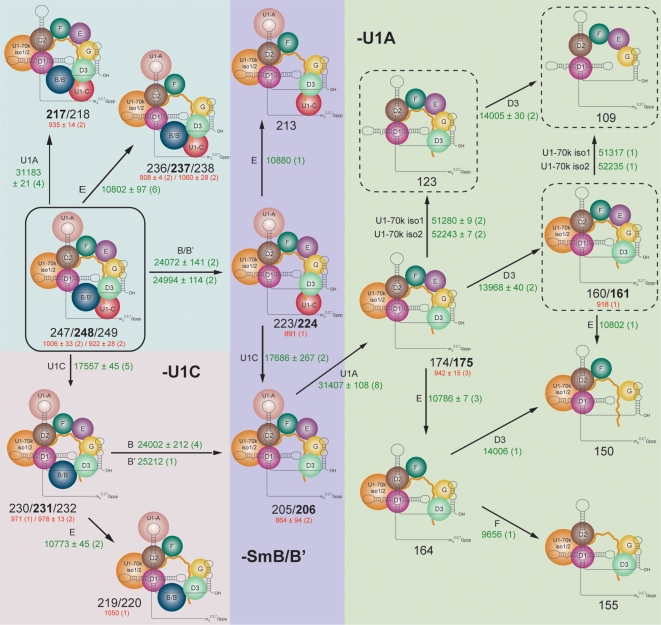
Summary of sub-complexes derived from U1 snRNP showing that the major dissociation pathways involve losses of U1-C, Sm-B/B', Sm-E and U1-A. Average experimentally-determined mass differences (green text with the number of spectra in parenthesis) acquired under a range of solution and MS conditions. Average experimentally-determined mass differences between the split peaks are given in red (number of spectra in parenthesis). Masses of each sub-complex are quoted in kDa (black text) with major species indicated in bold. Dotted borders indicate sub-complexes observed only as solution-phase species from the intact complex (solid border).

To generate additional sub-complexes to those formed in the gas phase we tried a number of different strategies employed previously, including addition of DMSO and *n*-butanol at 25% and 15% respectively [35], [48], as well as manipulation of the ionic strength [26]. Many solution conditions could not be used with this protein-RNA complex due to poor solubility and a tendency to precipitation. In 15% *n*-butanol and/or at lower ionic strength (128–150 mM ammonium acetate cf 200 mM Fig. 1, 2) spectra could be recorded that are consistent with further disruption of the complex (figure S2). The predominant species generated in solution (below the m/z values of the intact complex) are attributed to losses of Sm-B/B', U1-C and U1-A. Also curiously, these solution conditions promote the dissociation of U1-70k not readily observed in complexes formed by gas phase dissociation (figure 3: 123 and 109 kDa, table S6). The charge state series assigned to U1-A (+12/+13) is lower than that observed for gas phase dissociation (figure 1: +17/+18) consistent with dissociation occurring in solution. By contrast however charge states of the Sm-B/B', released in these solution disruption experiments (average 24/25+ for a 24 kDa protein) are greater than those observed following gas phase dissociation (cf ∼18+ for Sm-B/B', figure 1). Since charge states in electrospray are known to be highly dependent upon the conformation of the protein or complex [47] an average of one proton per kilodalton is usually attributed to an unfolded conformer. These solution conditions therefore induce dissociation of U1-A, without significant unfolding, and formation of highly charged Sm-B/B', consistent with its complete unfolding.

Major products formed both in solution and gas phase dissociation experiments, shown schematically using the crystal structure of the reconstituted core complex [17], reveal several common features (figure 3 and tables S7–8). The first point to note is that U1 snRNA is present in all the dissociation products, consistent with its multiple electrostatic interactions with the Sm core. Also of significance is the observation that loss of either Sm-B/B', U1-A, U1-C or Sm-E represents the first step in all major dissociation pathways. These results are surprising given previous observations, that in general the smallest subunits on the periphery of the complex are the most favourable to unfold and dissociate in the gas phase [24], [26]. We would therefore anticipate, based on mass alone, preferential loss of Sm-G (8.4 kDa) and Sm-F (9.6 kDa). While ready loss of Sm-E (10.7 kDa) is not unexpected given that its size is comparable to the smallest subunits, Sm-G appears remarkably stable being present in all 14 dissociation products. Interestingly, Sm-D3 also remains associated even when the ring is disrupted in more than one location (loss of Sm-F/E and Sm-B/B'). Its interactions with neighbouring Sm-G, and in turn with the N-terminus of U1-70k, as observed in the crystal structure, must therefore be sufficient to stabilise Sm-D3 within the fragmented core.

### The C-terminus of Sm-B/B' promotes its unfolding

**Figure 4:**
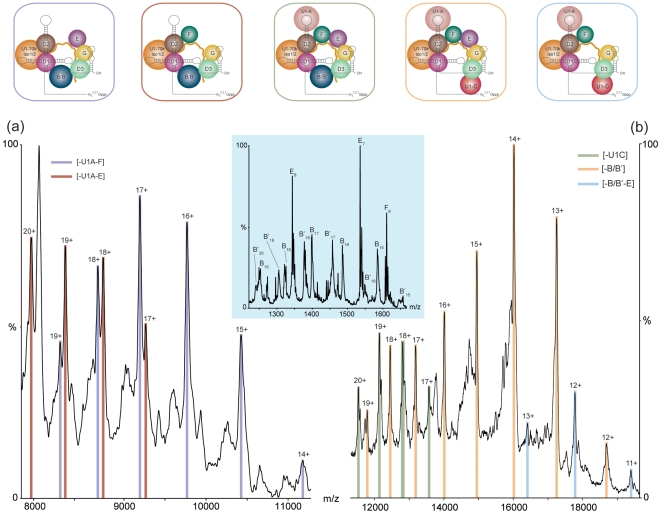
Comparison of spectra recorded for the U1 snRNP isolated from HeLa cells and a recombinant human U1 snRNP complex reconstituted *in vitro*. (a) The high m/z region of recombinant U1 snRNP reconstituted with all subunits except U1-C and with truncated Sm-B/B', shows remaining stripped complexes after loss of U1-A, Sm-E and Sm-F (or Sm-G* - masses of Sm-F and tagged recombinant Sm-G* cannot be distinguished (table S7) (b) U1 snRNP from HeLa cells showing predominantly loss of Sm-B/B'. Inset shows the low m/z region containing an extended distribution of charge states from dissociated full-length Sm-B/B'. Solution and MS conditions (a) 850 mM ammonium acetate. LCT: capillary: 1.5 kV, cone: 200 V, extractor: 75 V, source readback: 8.4 mbar, ToF readback: 2.1×10^−6^ mbar. (b) 200 mM ammonium acetate. QToF2: capillary: 1.5 kV, cone: 200 V, extractor: 0 V, collision cell voltage: 160 V, source transfer region readback: 7.1×10^−3^ mbar, ToF readback: 1.3×10^−6^ mbar.

The most intriguing result observed here however is the dissociation of Sm-B/B' (23.7/24.7 kDa), U1-C (17.4 kDa) and U1-A (31.2 kDa) in preference to all other smaller subunits in the Sm ring (with the exception of Sm-E). Sm-B/B' and U1-A are approximately three and four times larger than Sm-G respectively. Sub-stoichiometric binding and ready dissociation of U1-A are common observations in many of our spectra, implying labile association of this subunit with the intact particle. Moreover, unfolding does not appear to be a prerequisite of its gas or solution phase dissociation (figure S2). By contrast Sm-B/B' and U1-C undergo significant unfolding and appear to adopt multiple conformations, prompting their ready dissociation from the U1 snRNP complex both in solution and gas phases. Increasing further the activation energy applied to the intact cellular complex generates greater dissociation of protein subunits and higher intensity series for stripped complexes (figure 4 right) compared with experiments at lower activation energies. Three charge state series are identified, assigned to loss of U1-C and Sm-B/B' and to loss of both Sm-B/B' and Sm-E. The series assigned to loss of Sm-B/B' extend across at least eight charge states, in two different distributions, implying a remarkable ability of Sm-B/B' to accommodate charge prior to dissociation. The Sm-B/B' released under these conditions has an average charge state of 17/18+, in accord with dissociation in the gas phase (figure 1) but distinct from the complete unfolding observed in solution (figure S2). Interestingly these results also highlight the formation of a surprisingly stable complex, that of a partly formed 6-membered ring.

To understand the properties of Sm-B/B' and U1-C that might explain their propensity to unfold and dissociate readily we considered the sequences of the full-length subunits (supplementary text S1). Since both subunits have high arginine content and multiple proline-rich repeats at their C-termini, that are often intrinsically unstructured [49], this propensity to dissociate likely arises from their C-terminal tails. Such an unstructured region with multiple highly basic residues, which has been implicated in binding to other factors [50], would be particularly susceptible to charging. In order to test this hypothesis we compared the dissociation properties of Sm-B/B' in the cellular complex with a recombinant complex, formed by pairwise co-expression and incorporating a truncated version of Sm-B/B' comprising residues 1-174. All other subunits in the recombinant complex, as well as the synthetic U1 snRNA, were full-length as in the wild type complex; the only exceptions being U1-C which was not present, U1-70k which comprised residues 1–216 and Sm-G* which contained a C-terminal tag (table S9). Since the truncated Sm-B construct is larger than the other Sm ring proteins, but does not include the multiple C-terminal proline/arginine rich repeats, it allows us to isolate the effects of the unstructured tail within the context of the recombinant functional core.

The reconstituted complex was activated in the gas phase to induce dissociation of subunits (figure 4 left). Interestingly, the resulting spectrum shows that the dominant stripped complex is no longer assigned to loss of Sm-B_1–174_. Rather, major peaks are assigned to loss of U1-A, Sm-E and Sm-F. This ready dissociation of U1-A is consistent with observations for the cellular complex. The observation that truncated Sm-B remains associated with the complex however is surprising but reproducible across a series of constructs, on two mass spectrometers and from a variety of different solution conditions. Furthermore this observation is contrary to our observations for the cellular complex. This implies that the absence of the C-terminal tail reduces the propensity for unfolding and dissociation of Sm-B/B'. The multiple proline/arginine-rich C-terminal repeats, present in the wild type but not the recombinant form, therefore promote unfolding and dissociation of Sm-B/B' in preference to the smaller Sm ring proteins. Our results strongly imply therefore that the C-terminal proline rich repeats are unfolded and exposed in the intact complex, acting to initiate unfolding and charging of Sm-B/B' in solution and gas phase experiments, thereby promoting their dissociation.

### U1-70k isoforms affect subunit interfaces

**Figure 5:**
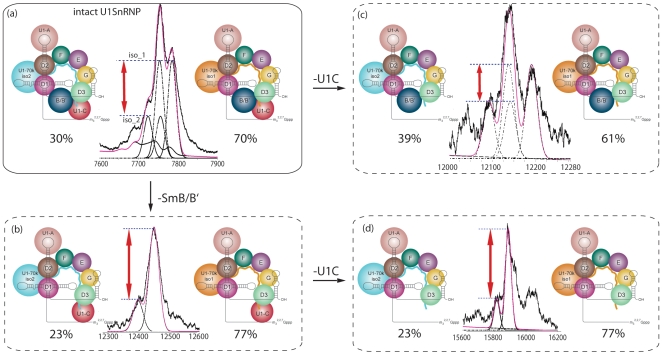
Monitoring changes in the ratio of the U1-70k isoforms in charge states of the intact spectrum and dissociation products. All peaks are scaled according to the ratio of the U1-70k isoforms(red arrows) to enable direct comparison irrespective of the presence of Sm-B/B'. The ratio of isoform 2: isoform 1 in the intact complex is (70∶30) (a). For loss of U1-C the isoform 2 : isoform 1 ratio decreases to 39∶61 (b) while loss of Sm-B/B' leads to an increase in the ratio 23∶77 (c). Subsequent loss of U1-C from the complex in which Sm-B/B' is absent reveals the same isoform ratio (23∶77) as observed for the loss of Sm-B/B'.

To determine whether or not the dissociation of subunits Sm-B/B' and U1-C is affected by the presence of different U1-70k isoforms we monitored the population of isoforms in the stripped complexes formed in the gas phase. The ratio of the two isoforms (U1-70k isoform 1 and isoform 2) in the intact complex was determined above as 70∶30 (figure S3 and figure 5a). At high activation energy dissociation of Sm-B/B' occurs with equal propensity from both U1-70k isoforms, the initial ratio is therefore maintained (70∶30) (figure S4). At lower activation energy, when Sm-B/B' is dissociated from the complex however an increase in the disparity between the two populations is observed (isoform 1: isoform 2, 77∶23, figure 5b and figure S5). This is an intriguing result which we interpret in terms of a reduced tendency for dissociation of Sm-B/B' from the U1-70k isoform 2. Turning our attention to loss of U1-C directly from the intact complex, rather than an increase in the ratio, we observe a clear decrease (61∶39 isoforms 1∶2, figure 5c and figure S6). This is attributed to a greater tendency for U1-70k isoform 1 to retain U1-C than isoform 2 (figure 5c). Considering sequential loss of Sm-B/B' followed by U1-C we find that the increased ratio (77∶23) is maintained in this product, consistent with our observation for loss of Sm-B/B' alone. Overall therefore we can conclude that the subunit interfaces between U1-C and Sm-B/B' are enhanced by interaction with U1-70k isoforms 1 and 2 respectively.

## Discussion

We have determined that the U1 snRNP complex, isolated directly from HeLa cells, contains four different protein isoforms. By defining the extent of incorporation of splice variants of U1-70k and Sm-B/B' we were able to probe their effects on the overall stability of the various sub-complexes. We have also generated and assigned 14 different sub-complexes, formed by perturbation in solution or dissociation in the gas phase. Comparison of the subunit composition of these sub-complexes implies highly stable interfaces between both isoforms of U1-70k with Sm-D1 and Sm-D2. By contrast, facile losses of Sm-E, U1-C and Sm-B/B' are common to all dissociation products and lead to disruption of the ring on opposite sides, revealing stable interactions of Sm-G with Sm-D3 and U1 snRNA. Moreover, given that neither U1-A or U1-C are retained in sub-complexes in which U1-70k is absent, our results also suggest that U1-70k provides an anchor, not only for the core interactions with Sm-D1/D2, but also for longer range interactions with either one or both of the other U1 snRNP specific proteins. Strikingly, this observation is entirely consistent with the crystal structure of U1 snRNP where it is observed that the N-terminus of U1-70k crosses Sm-D2, near its interface with Sm-D1 and physically acts to stabilize the incorporation of U1-C [17].

Among the most surprising findings of our study are the observations that both Sm-B/B' and U1-C are lost readily in the gas phase and prior to the smaller subunits (Sm-G, Sm-F, Sm-D1, Sm-D2 and Sm-D3). This implies an ease of unfolding for Sm-B/B' and U1-C compared with the other Sm proteins. Our solution disruption experiments reinforce this view by showing that the Sm-B/B' subunits unfold more readily than any other subunit in the Sm ring, giving rise to an average of 23/24+ charges for the 26 kDa protein. Interestingly, this is very similar to the extent of charging observed in mass spectra reported previously for full-length U1-C (17.3 kDa). In this case average charge states of 9+ and 17+ were observed for folded and unstructured conformers respectively [51]. In the same study, an N-terminal construct of U1-C, comprising residues 1–61, had relatively few charges (5+), was shown to bind Zn^2+^ and to interact with reconstituted U1 snRNP in which U1-C was omitted [51]. Together these results strongly imply that the first 61 resides from the N-terminus are folded. Comparison of the charge states of U1-C_1–61_ (5+) with the highly charged conformations observed for full length U1-C (17+) however suggests that the C-terminal tail of the full-length protein is unfolded in solution and consequently more accessible to charging. This is analogous to the situation for full length Sm-B/B' and a truncated form of Sm-B, which did not undergo sufficient charging to promote its unfolding and dissociation from the recombinant complex. Therefore we conclude that both Sm-B/B' and U1-C undergo facile unfolding in solution and gas phases due to the presence of their intrinsically unstructured C-terminal tails.

If we consider the assembly pathway of the U1 snRNP *in vivo* which occurs in the cytoplasm after transcription and export of the snRNA from the nucleus, the Sm core assembly begins with the formation of the Sm ring around the Sm site [8], [52], [53]. Interestingly, the first stable sub-complex in the assembly pathway of U1 snRNA: Sm-D1: Sm-D2: Sm-E: Sm-F: Sm-G is also a predominant species formed in our solution disruption experiments suggesting, as was observed recently for homomeric proteins, that the disassembly of protein complexes in solution often recapitulates their assembly pathway *in vivo* [48]. The next step in assembly is binding of the Sm-D3:Sm-B/B' heterodimer. In our experiments the fully assembled Sm ring in the absence of the U1 snRNP specific proteins is not observed as a stable sub-complex. Under the conditions of our experiments full length U1-C and Sm-B/B' are too labile to remain associated with the Sm ring in the absence of U1-70k.

Given our observation that the stability of subcomplexes is not affected by incorporation of full length Sm-B/B', it is likely that the main function of SmB/B' is not in stabilising the ring but rather in providing a platform for additional factors, binding to the unstructured C-terminus and enabling them to modify the fully formed core. Interestingly Sm-B/B' has been shown to be important for bringing at least one additional factor to the spliceosome, the trimethyl guanosine synthase [54]. This protein cofactor attaches to the C-terminal end of the Sm-B/B', once this subunit is integrated into the Sm ring, and is responsible for transferring two methyl groups to the m_7_G cap of the snRNA [54]. Our results strongly suggest that this factor binding to Sm-B/B', together with the SMN complex confers the necessary stability to the Sm ring such that it survives within the cytoplasm in the absence of the U1 specific proteins. Following interactions of SMN with importin β, nuclear import takes place coupled with dissociation of SMN, modifications to the snRNA and binding of U1-A, U1-C, and U1-70k. The subsequent binding of these U1 snRNP specific proteins likely confers the independent stability to the Sm ring, such that it can be isolated intact and necessary for the final maturation events that are essential for function.

**Figure 6:**
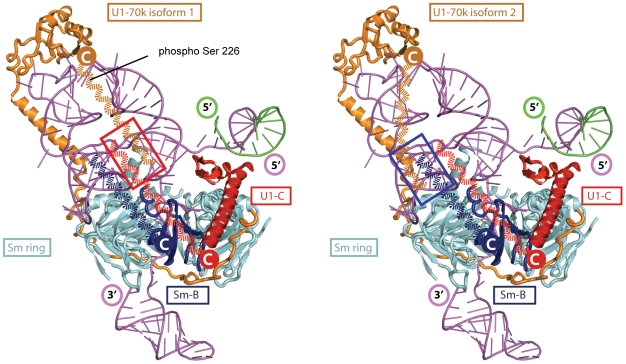
A model of interactions between the C-terminal tails of U1-70k isoforms 1 (left) and 2 (right) with U1-C and Sm-B/B', respectively. Possible path of the C-terminal tails of two U1-70k isoforms drawn approximately to scale (dashed curved orange lines) and modeled onto the crystal structure of human U1 snRNP [17] and interactions between C-terminal tails of U1-C and SmB/B' are highlighted (red and blue boxes respectively). Indicated are: U1 snRNA (magenta); Sm-B/B' (blue); Sm-D1, D2, D3, E, F and G (cyan); U1-70k (orange); U1-C (red); speculative path of the C-terminal tails of U1-70k isoforms 1 and 2 (dashed orange lines).

One of the important insights gained from our study is the observation that U1-70k isoform 2 binds more stably to Sm-B/B' than isoform 1 while the converse is true for U1-C. This is an intriguing result given the close sequence similarity that exists between the two U1-70k isoforms, the only difference being an additional 9 amino acids (residues 223–231) for isoform 1. Interestingly however this sequence incorporates Ser_226_ shown in our proteomics experiments to be phosphorylated (figure S7) in line with previous reports [43], [46]. This therefore implies that the combination of the extra length of protein chain as well as the additional phosphorylation site enhances interactions with U1-C (figure 6). In contrast, the shorter version of U1-70k (isoform 2), with one less phosphorylation site, increases interactions with Sm-B/B'. It is established crystallographically that the N-terminus of U1-70k is extended and wraps around the ring to contact U1-C [17]. This path begins just N-terminal to its RDB, the motif that mediates its interaction with a stem-loop of U1 snRNA [17]. The additional amino acids in U1-70k isoform 1 occur C-terminal to the structured RBD and are also predicted to be unstructured [17]. Given our results that show that the C-terminal tails of the two U1-70k isoforms interact differently with Sm-B/B' and U1-C, on the opposite side of the ring to the extended N-terminus, and considering the high proportion of acidic residues in the C-terminal 45 residues (22% cf 4% basic) these unstructured acidic residues are poised for interaction with complementary basic groups. Given the highly basic arginine-rich C-terminal sequences of Sm-B/B' and U1-C and the fact that the C-terminus contains all but one of the six phosphorylation sites reported for U1-70k, it is entirely feasible that phosphorylation is responsible for fine tuning the interactions of the negatively charged C-terminal tails with the unstructured regions of positively charged Sm-B/B' and U1-C. This allows us to propose that an important role of U1-70k is in fine tuning interactions with either U1-C or Sm-/B/B' in response to incorporation of the different isoforms and their phosphorylation status *in vivo*.

It is also interesting to consider interactions of the different isoforms and their likely proximity to additional cofactors and subunits. Since we find no preference for interaction of the longer version of U1-70k with the longer splice variant of Sm-B we speculate that the role of Sm-B/B' is more likely to be in differential interactions with protein cofactors. The observation that the Sm-B/B' isoforms are present in a 1∶1 ratio while the longer isoform of U1-70k is preferred raises interesting evolutionary questions. One explanation is that in addition, and analogous to the interactions of Sm-B/B', the C-terminal tail of U1-70k may have evolved for optimal binding to numerous constitutive and alternative splicing factors [55]. The U1-70k isoforms 1 and 2 could therefore promote different “modes” of splicing, altering quaternary structures by strengthening interactions with U1-C and SmB/B' respectively. Additionally they may also switch quaternary structure and “mode” in response to phosphorylation of the additional serine residue present in isoform 1. Interestingly, the interaction site of U2 snRNP was recently located close to the proposed location of the C-terminal tails of SmB/B', U1C and U1-70k in our model (figure 6) [56]. Consequently this switch in structure in response to phosphorylation is also likely important for promoting interactions between U1 snRNP and U2 snRNP, a critical stage in spliceosome assembly.

In summary, our structural model identifies many of the dynamic subunits, not revealed by other structural techniques, that provide an important additional layer of complexity to the quaternary structure of human U1 snRNP. More generally, our results present a methodology for the study of subunit architecture of functional multiprotein complexes by revealing mechanistic insights into the role of intrinsically unstructured regions and post-translationally modified subunits within the context of a cellular machine.

## Methods

### Purification of U1 snRNP from HeLa cells

Rabbit polyclonal antibodies (Eurogentec) were raised against a peptide (aa 1–14) of the human U1-A protein and affinity purified using a SulfoLink column (Pierce) containing the antigenic peptide. 600 µl of Protein A Sepharose (GE Healthcare) were charged with 360 µg of affinity purified antibodies and preblocked with 0.3 mg/ml BSA and 50 µg/ml yeast tRNA. Immunoprecipitations (IP) were carried out in 6 tubes, each containing 100 µl of PAS, 500 µL of IP150 buffer (20 mM Hepes, pH 7.9, 150 mM NaCl, 1.5 mM MgCl2, 0.5 mM DTT) and 500 µl of HeLa nuclear extract in buffer C prepared according to [57]. The tubes were incubated with head-over-tail rotation for 3 h at 4°C. Then, each aliquot of beads was washed six times with 1 ml of IP150 and eluted in 400 µl of the same buffer containing 0.6 mg/ml antigenic peptide. The eluate (6×400 µL) was loaded on six 4 ml 5–20% glycerol gradient in IP150 buffer, centrifuged for 14 h at 37 000 rpm in TH-660 rotor (Sorvall), and fractionated manually into 24 175-µl fractions. Fractions 10–14 containing the U1 snRNPs were combined and snRNPs were concentrated by pelleting for 6 h at 60 000 rpm in TH-660 rotor (Sorvall). The pellet was re-suspended in 150 µl of 20 mM HEPES, pH 7.9, 150 mM KCl, 0.5 mM DTT, 5% glycerol by occasional pipetting the sample and keeping it on ice for 30 min. Aliquots of 30 µl were frozen and kept at −80°C. The integrity of the U1 snRNPs after dialysis against 0.2 M of ammonium acetate was confirmed by gradient density centrifugation carried out under conditions described above.

### Separation of proteins and RNA

To the sample of U1 snRNPs (approximately 50 pmoles) ammonium acetate and SDS were added to 0.3 M and 0.5% correspondingly. 500 µl were extracted with an equal volume of acidic phenol (Sigma) followed by extraction with phenol/chloroform/isoamyl alcohol (25∶24∶1). The U1 snRNA was precipitated from the aqueous phase by addition of 2.5 volumes of ethanol, re-suspended, divided into five aliquots, precipitated with ethanol and stored as a dry pellet. The proteins were precipitated from the phenol phase by addition of five volumes of acetone (five aliquots).

### Purification and preparation of reconstituted human U1 snRNP

Preparation of RNA and U1 snRNP protein subunits and the reconstitution of the human U1 snRNP complex, was carried out as reported previously [17], [30].

### Buffer exchange of the cellular U1 snRNP complex

The final purification buffer (20 mM HEPES pH 7.9, 150 mM KCl, 0.5 mM DTT and 5% glycerol) was exchanged to ammonium acetate using drop dialysis or centrifugal ultrafiltration. Drop dialysis of a 30 µl aliquot was carried out overnight at 4°C using a VSWP filter disc (13 mm, 0.025 µm, Millipore). For centrifugal ultrafiltration, a 35 µl aliquot was buffer exchanged using a Vivaspin 0.5 ml device (10 kDa MWCO) at 12000 g and 4°C to a dilution factor (original buffer) of 7.5×10^5^. 200 mM ammonium acetate solution was used for buffer exchange.

### Buffer exchange of the recombinant complex

The recombinant U1 snRNP was purified off an anion-exchange (MonoQ) column using a sodium chloride gradient in a neutral pH solution (20 mM HEPES, pH 7.5). Peak fractions were pooled and the sample (generally 25 µl in volume) was dialyzed against 0.25–1.0 M ammonium acetate which has a pH ∼7. To dialyze small volumes samples were placed in a dialysis button, one face having a dialysis membrane of MWCO 10–12 kDa. The dialysis button was placed in a 50 ml falcon tube containing ammonium acetate and rocked overnight at 4°C.

### Nano ESI-MS

All data were acquired using standard nano ESI interfaces and either a Q-ToF2 mass spectrometer, modified for high m/z acquisition of non-covalent complexes [58], or an LCT mass spectrometer with an additional gas inlet in the transfer hexapole (Waters, Manchester UK). Samples were loaded into borosilicate capillaries, 1.0 mm o.d.×0.5 mm i.d. (Harvard Apparatus, Edenbridge, U.K.), which were drawn down to a fine taper, coated with gold and cut manually under a stereomicroscope to give the required diameter and flow.

Collisional cooling was achieved through adjustment of the source rotary pump isolation valve to give the required pressure in the source and transfer hexapole regions. Argon was used as collision gas in the collision cell (QToF2)/transfer hexapole (LCT). Other experimental details have been described previously [59]. Specific MS conditions are included in table S3.

## Supporting Information

Text S1 Supplementary Text S1
(0.06 MB DOC)

Figure S1 LC/UV (a) and LC/ESI-MS (b) chromatograms from U1 snRNP isolated from HeLa cells. The peak height ratio of Sm-B:Sm-B'in the UV (214 nm) trace is measured as 0.96. Using either only the number of amide bonds or calculated extinction co-efficients for amide bonds and all amino acid residues (Kuipers and Gruppen, 2007) gives a Sm-B:Sm-B' concentration ratio of 1.0. Protein identities were determined by LC/MALDI using on-plate tryptic digestion (see supplementary text). Protein identities: (1) Sm-D3, (2) Sm-B/B' fragment [13438 Da], (3) U1-C, (4) Sm-B', (5) Sm-B, (6) Sm-F, (7) Sm-G and U2 snRNP-A' [28284 Da], (8) U2 snRNP-B' [25529 Da], (9) U1-A, (10) Sm-E Peak 3 (Sm-B/B' fragment) was only observed in spectra from acidic solution conditions. The two U2 snRNP proteins were present as contaminants from purification.
(0.24 MB PDF)

Figure S2 Electrospray mass spectra of the cellular U1 snRNP after buffer exchange to 150 mM ammonium acetate using centrifugal ultrafiltration followed by addition of butanol to give 128 mM ammonium acetate with 15% (v/v) butanol solution. Similar spectra were obtained from 150 mM ammonium acetate solution with no butanol present. The intact complex was not detected under these conditions. Predominant gas-phase dissociation of U1-C, Sm-B/B' and U1-A (pink inset) are observed together with two solution phase sub-complexes (blue inset) due to loss of all three U1-specific proteins together with Sm-B/B' and Sm-D3. At low m/z, Sm-B/B' is observed at unusually high charge states (green inset), consistent with an unfolded subunit. MS conditions: capillary: 1.3 kV, cone: 99 V, extractor: 100 V, collision cell voltage: 80 V, source readback: 3.4 mbar, analyser readback: 2.9×10−4 mbar, ToF readback: 1.1×10−6 mbar
(0.33 MB PDF)

Figure S3 Fit of the intact U1snRNP complex. Fit of the U1snRNP complex with 4 Gaussians representing the different isoforms of subunit B/B' and U1-70k_1/_2. On the left the best fits for the different charge states of the complex are shown. The middle column shows the error function for the fit of the peak heights of the first peaks. The column on the right represents the fit for the abundance ratio of the U1-70k_1 isoform. The determined abundance is 30.2% : 69.8%.
(0.37 MB PDF)

Figure S4 Higher energy activation: effect of product isoform ratio. Simulation of the peaks corresponding to the intact U1 snRNP containing different isoforms of SmB/B' and U1-70k isoforms 1 and 2. By monitoring the change in ratio of the peaks during dissociation of subunits we can assess potential interactions with various subunits. Under high activation conditions (collision cell voltage 160 V) the ratio of the U1-70k isoforms 1 and 2 however is indistinguishable from that in the intact complex. This in contrast to our results at lower activation conditions (collision cell 100 V) (figure 5 main text) where clear differences are observed in the ratio of the two isoforms after dissociation of Sm-B/B' and U1-C.
(0.48 MB PDF)

Figure S5 Fit of the -[U1-C] complex. Fit of the U1snRNP complex from which U1C has dissociated. The best fits obtained by minimizing the error of the fit and the spectra are shown (left). The error associated with the fitting is shown in the middle column. The abundance determined is 38.6% : 61.4% for U1-70k isoform 2:U1-70k isoform 1. For comparison three spectra on the right show Gaussian peaks representing the profile that would be obtained if there was no change in abundance with the peak height being optimized for the first, second or third peak from left to right respectively.
(0.34 MB PDF)

Figure S6 Fit of the -[B/B'] complex. Fit of the U1snRNP complex from which B/B' has dissociated. The best fits obtained by minimizing the error of the fit and the spectra are shown in the first column (left). The abundance determined is 23.3% : 76.7%. The error of the fits is shown in the middle column. For comparison the column on the right shows Gaussian peaks representing the distributions that would occur, if no change in the ratio of isoform abundance took place.
(0.35 MB PDF)

Figure S7 Phosphorylation of Ser226 in U1-70k isoform 1. Tandem MS spectra of the peptide Y_219 to R_231 which encompass the additional residues in U1-70k isoform 1 (a) and its phosphorylated from (b) recorded on the LTQ-Orbitrap after LC separation. Insets in (a) and (b) summarize series of b and y ions identified for these two peptides allowing confident assignment of their sequences and identification of a phosphorylation at Serine226. The tryptic digest was separated on a Ultimate 3000 HPLC system (Dionex) using a nanoC18 column with a 75 um i.d.. 0.1% formic acid was added to the mobile phase and the gradient was 0–45% acetonitrile in 30 minutes at a flow rate of 0.3 uL/min. Under these conditions the retention times for the phosphorylated and non-phosphorylated forms were 15.07 and 15.11 min.,respectively. Mass spectrometric analyses were performed using a hybrid LTQ orbitrap mass spectrometer (Thermo Fischer Scientific). Nano ESI was initiated by applying 1.85 kV to the picotip. The ion transfer capillary voltage and temperature were 35 V and 275°C respectively. The tube lens voltage was set to 110 V. External calibration was performed using the manufacturer's calibration mix. MS/MS was carried out using helium as collision gas and 6 scans were performed in the ion trap for the 6 most intense peaks per full scan at a normalized collisional energy of 35V and a maximum injection time of 100 ms.
(0.31 MB PDF)

Table S1 Experimental and calculated masses of U1 snRNP proteins and RNA from HeLa cell complex
(0.04 MB DOC)

Table S2 Calculated masses of intact U1 snRNP and sub-complexes.
(0.03 MB DOC)

Table S3 Masses of complexes, subcomplexes and single proteins.
(0.14 MB DOC)

Table S4 Reported phosphorylation sites in U1-70k from HeLa cells.
(0.05 MB DOC)

Table S5 Parameters used to model experimental spectra. The mass shifts were added to the theoretical masses of the (sub)complexes prior to modeling. This enabled matching of the mean of each peak Gaussian with the corresponding peak top in the experimental spectrum. These mass shifts are due to buffer, water and salt molecules that adhere to the protein complex and were found to be of a lower value for the CID complexes than for the solution phase complexes. Varying resolution of the peaks as well as different adducts lead to broad peaks in the spectra which is reflected in their different full width at half maximum (FWHM) values.
(0.03 MB DOC)

Table S6 Possible compositions of 109 kDa and 123 kDa subcomplexes
(0.04 MB DOC)

Table S7 Dataset of masses used for input in SUMMIT.
(0.03 MB DOC)

Table S8 Average mass differences and protein identities.
(0.04 MB DOC)

Table S9 Experimental masses of U1 snRNP proteins and RNA from the recombinant complex.
(0.03 MB DOC)
