# Supplementary material for: Isoforms of U1-70k Control Subunit Dynamics in the Human Spliceosomal U1 snRNP
Source: PLoS One. 2009 Sep 28;4(9):e7202. doi: 10.1371/journal.pone.0007202 (PMC2747018; doi:10.1371/journal.pone.0007202)
Supplement: Text S1 — Supplementary Text S1 (0.06 MB DOC) [file pone.0007202.s001.doc]

**Supplementary Text**

**Experimental methods**

**Separation of U1 snRNP protein from U1 snRNA for MS analysis**

To the sample of U1 snRNPs from HeLa cells (approximately 100 pmoles) ammonium acetate and SDS were added to 0.3 M and 0.5% correspondingly. 500 µl of the sample were extracted with an equal volume of acidic phenol (Sigma) followed by extraction with phenol/chloroform/ isoamyl alcohol (25:24:1). U1 snRNA was precipitated from the aqueous phase by the addition of 2.5 volumes of ethanol, resuspended again, divided into five aliquots, precipitated with ethanol and kept as a dry pellet. The proteins were precipitated from the phenol phase by addition of five volumes of acetone.

An aliquot of U1 snRNA was resuspended with 20 µl 1M ammonium acetate (mass spectrometry grade, Fluka) prepared with filtered, autoclaved water, and vortexed briefly prior to desalting (in order to remove Na^+^ introduced by the earlier precipitation step) using two autoclaved MicroBiospin-6 centrifuge columns and 1 M ammonium acetate buffer.

**Lyophilised U1 snRNP from HeLa cells**

Following isolation in buffer containing: 20 mM HEPES pH 7.9, 150 mM KCl, 0.5 mM DTT and 5% glycerol, approximately 50 pmoles of U1 snRNP was dialysed for 3 h against 5% acetic acid and then overnight against 1% acetic acid using a Slide-A-Lyser before lyophilisation. The sample was resuspended in 40 µl 1% acetic acid and divided into 5 µl aliquots (12.5 pmoles each) which were lyophilised and stored as dry pellets at -20 °C.

**Proteomic analysis of U1 snRNP from HeLa cells**

The proteins present following purification of U1 snRNP were identified by tryptic digestion. A 12.5 pmole aliquot of lyophilised U1 snRNP (see above) was dissolved in 200 mM ammonium bicarbonate/ 4 M urea containing 12.5 µg/mL trypsin (modified sequencing grade, Promega) and digested overnight at 37 °C. The digest was desalted using a C18 Zip-Tip / 0.1% formic acid in 50 % acetonitrile prior to analysis by LC/MS/MS using a LC-Packings Ultimate System (Dionex, Sunnyvale, CA fitted with a 75 μm i.d. x 15 cm C18 PepMap100 (3 μm) nano-LC column run at a flow rate of 300 nl/min. Peptides were eluted using a linear gradient of 0 - 50% B over 30 min. where solvent A: 0.05%(v/v) trifluoroacetic acid (TFA) in 100% water and solvent B: 0.04% (v/v) TFA in 90% acetonitrile. The LC was interfaced to a LTQ Orbitrap mass spectrometer (Thermo Fischer Scientific). Nano ESI was initiated by applying 1.85 kV to the picotip. The capillary voltage and temperature were 35 V and 275 °C respectively. Tube lens voltage was set to 110 V. External calibration was performed using the manufacturer’s calibration mix. MS/MS were carried out using helium as collision gas and 6 scans were performed in the ion trap per full scan at a normalized collisional energy of 35 V and a maximum injection time of 100 ms. All MS/MS samples were analyzed using the Sequest search engine (Thermo Finnigan). Sequest was set up to search the National Center for Biotechnology Information database assuming the digestion enzyme trypsin. Mascot was searched with a fragment ion mass tolerance of 0.50 Da and a parent ion tolerance of 10 ppm. All proteins identified had cross correlation */scores/* (Xcorr) above 20 and contained at least 3 identified peptides.Peptides were obtained for all expected U1 snRNP proteins (Sm-D1, Sm-D2, Sm-D3, Sm-B/B', Sm-E, Sm-F, Sm-G, U1-70k, U1-A and U1-C) and additionally peptides from two U2 snRNP proteins: A' (Uniprot accession code P09661) and B'' (Uniprot accession code P08579).

**LC/UV and LC/ESI**

The masses of individual subunits were measured following LC separation of the proteins. A LC-Packings Ultimate System (Dionex, Sunnyvale, CA) with a capillary UV detector (214/280 nm) was interfaced to a QSTAR Elite mass spectrometer (Applied Biosystems) fitted with a standard ESI source. Separation was achieved using a capillary PS-DVB monolithic column (200 um i.d x 5 cm) held at 60 °C with a flow rate of 3 µl/min and a linear gradient of 10 - 70 % solvent B over 20 mins, where solvent A: 0.05% (v/v) trifluoroacetic acid (TFA) in 100% water and solvent B: 0.04% (v/v) TFA in 90% acetonitrile. Column loading was approximately 0.5 pmole of each subunit (1 µl injection volume from a 12.5 pmole lyophilised aliquot resuspended in 24 µl 1% (v/v) formic acid).

Chromatograms are shown in figure S1 and the experimental masses summarized in table S3. Four proteins were not detected: U1-70k isoform 1, U1-70k isoform 2, Sm-D1 and Sm-D2.

**LC/on-plate digestion/MALDI MS/MS**

Protein identities were correlated with their masses by another LC analysis (using the same separation conditions as LC/UV/ESI) with the column eluate directed to a Probot sample fraction system (Dionex) for spotting onto a MALDI plate. Automated spotting was carried out at 10 s intervals (0.5 µL) onto a MALDI plate prespotted with 150 ng/µL trypsin (sequencing grade, Promega) in water. When dry, the sample spots were overlaid with 0.7 µL 25 mM ammonium bicarbonate, digested for 10 min in a humidifier, vacuum-dried and overlaid with 0.5 µL α-cyano-4-hydroxycinnamic acid (5 mg/ml in 1:1 water:acetonitrile with 0.1%(v/v) TFA) matrix before analysis by automated MALDI-TOF/TOFMS/MS (Applied Biosystems 4700). Data-dependent peak selection of the three most abundant MS ions was used for CID. Ar was used as the collision gas. Resulting data were analyzed by GPS Explorer (Applied Biosystems), which involved a MASCOT (Matrix Science) database search using all entries in the NCBI database to determine candidate peptides. Mass tolerance for the precursor ion was set to 100 ppm; mass tolerance for the fragment ions was 0.3 Da.

In agreement with the LC/ESI results, peptides from four proteins (shown to be present by tryptic digestion in urea) were not detected: U1-70k isoform 1, U1-70k isoform 2, Sm-D1 and Sm-D2.

**Data analysis**

**Assignment of the subcomplexes**

The complexes generated by gas phase dissociation or solution phase disruption were assigned using mass differences. An iterative algorithm, SUMMIT was employed which calculates all possible combinations of subunits that sum to the mass determined experimentally (Taverner et al., 2008). The number of copies of each sub-unit was constrained to a maximum of one, given our determination of stoichiometric binding for each of the ten proteins. The masses of the subunits are listed in dataset 1 (table S5). There are cases where assignment of the mass differences between complexes is ambiguous (e.g. 31 kDa and 24 kDa) since these values can be assigned to single subunits (U1-A or Sm-B/B' respectively) or to masses of two or more sub-units (eg U1-C:D_2_ or Sm-E:D_2_). Assignments involving simultaneous loss of two or more subunits however are rare for complexes generated by collision induced dissociation and usually involve an unusual stability (Aquilina, 2008; Benesch et al., 2006; van den Heuvel et al., 2006). We therefore restricted our dataset to stepwise losses of individual subunits and were able to make assignments with reasonable confidence by considering series of subcomplexes related by the loss of single subunits.

**Simulation**

.

The experimental spectrum consists of overlapping peak series of intact U1 snRNP and several subcomplexes. These were simulated separately and summed together with a simulated background to resemble the experimental data as closely as possible using a program written in-house based on Labview. Due to the attachment of buffer salts, water and solvent the experimental data shows peak broadening and a mass shift of the peak centres towards higher masses (table S1). This shift depends mainly on the conditions which the complexes experience. It is therefore similar for all the complexes in one spectrum that are formed by dissociation in solution. A different value that is slightly smaller than for solution phase complexes is applied to the subcomplexes formed in the gas phase by CID. The program allows adjustment of the mass shift of the complexes and the width of the Gaussians accordingly.

Peaks belonging to one complex are modeled by a distribution of three parameter Gaussians, with the observed m/z values as mid points. The peak widths are adjusted according to the broadness of the experimental peaks. The change of signal intensities for the different charge states follows a Gaussian distribution (Sobott et al., 2002). Therefore the peak heights for the different charge states of the components are scaled by a Gaussian distribution, with parameters fitted to each peak series. The two isoforms of the subunits U1-70k and Sm-B/B' lead to peak splitting, which is taken into account by fitting the resulting peak distribution to four or two Gaussians per charge state depending on whether or not Sm-B/B' is present in the subcomplex (see insets in figure 2). We assumed that the relative response of the peaks from a particular subcomplex remained constant over all its charge states; the fit obtained between experimental and simulated data demonstrated that for our data this was a valid approach.

The component simulations are summed and parameters optimized to achieve maximal agreement of the summed simulation with the experimental mass spectra. (see figure 2).

The separate simulations allow determination of the area under all peaks belonging to one complex. These values are used to calculate the relative intensity between the different species.

**Calculation of the errors associated with the fit**

The peak height of the first peak (isoform U1-70k_2) of a doublet / triplet is determined by minimizing the deviation between the experimental spectrum from the Gaussian peak fit. The error of the fit is then calculated with the least square method. Since peak broadening, due to attachment of water/buffer molecules distorts the peak shape on the high m/z side, only the top part of the peaks (broadness FWHM/2) is used for the error calculations. (see above). The abundances of the two isoforms are determined by fitting one / three additional Gaussians to the experimental peaks, varying the percentage distribution between the two isoforms and thereby the peak heights of the complexes containing isoform U1-70k_1. This is optimized by minimizing the error of the fit, which is calculated using the least square method. Examples for the fits as well as examples for fitted peak multiplets are shown in figure 4A-C.

**Isoform sequences**

Differences between isoforms highlighted in red.

Construct Sm-B (1-174) indicated in blue.

U1-70k RNA binding domain (92-202) indicated in green.

**Sm-B (Swiss-Prot: P14678-2)**

MTVGKSSKMLQHIDYRMRCILQDGRIFIGTFKAFDKHMNLILCDCDEFRKIKPKNSKQAEREEKRVLGLVLLRGENLVSMTVEGPPPKDTGIARVPLAGAAGGPGIGRAAGRGIPAGVPMPQAPAGLAGPVRGVGGPSQQVMTPQGRGTVAAAAAAATASIAGAPTQYPPGRGGPPPPMGRGAPPPGMMGPPPGMRPPMGPPMGIPPGRGTPMGMPPPGMRPPPPGMRGLL

**Sm-B' (Swiss-Prot: P14678-1)**

MTVGKSSKMLQHIDYRMRCILQDGRIFIGTFKAFDKHMNLILCDCDEFRKIKPKNSKQAEREEKRVLGLVLLRGENLVSMTVEGPPPKDTGIARVPLAGAAGGPGIGRAAGRGIPAGVPMPQAPAGLAGPVRGVGGPSQQVMTPQGRGTVAAAAAAATASIAGAPTQYPPGRGGPPPPMGRGAPPPGMMGPPPGMRPPMGPPMGIPPGRGTPMGMPPPGMRPPPPGMRGPPPPGMRPPRP

**U1-70k isoform 1 (U170k_1) (Swiss-Prot: P08621-1)**

MTQFLPPNLLALFAPRDPIPYLPPLEKLPHEKHHNQPYCGIAPYIREFEDPRDAPPPTRAETREERMERKRREKIERRQQEVETELKMWDPHNDPNAQGDAFKTLFVARVNYDTTESKLRREFEVYGPIKRIHMVYSKRSGKPRGYAFIEYEHERDMHSAYKHADGKKIDGRRVLVDVERGRTVKGWRPRRLGGGLGGTRRGGADVNIRHSGRDDTSRYDERPGPSPLPHRDRDRDRERERRERSRERDKERERRRSRSRDRRRRSRSRDKEERRRSRERSKDKDRDRKRRSSRSRERARRERERKEELRGGGGDMAEPSEAGDAPPDDGPPGELGPDGPDGPEEKGRDRDRERRRSHRSERERRRDRDRDRDRDREHKRGERGSERGRDEARGGGGGQDNGLEGLGNDSRDMYMESEGGDGYLAPENGYLMEAAPE

**U1-70k isoform 2 (U170k_2) (Swiss-Prot: P08621-2)**

MTQFLPPNLLALFAPRDPIPYLPPLEKLPHEKHHNQPYCGIAPYIREFEDPRDAPPPTRAETREERMERKRREKIERRQQEVETELKMWDPHNDPNAQGDAFKTLFVARVNYDTTESKLRREFEVYGPIKRIHMVYSKRSGKPRGYAFIEYEHERDMHSAYKHADGKKIDGRRVLVDVERGRTVKGWRPRRLGGGLGGTRRGGADVNIRHSGRDDTSRYDERDRDRDRERERRERSRERDKERERRRSRSRDRRRRSRSRDKEERRRSRERSKDKDRDRKRRSSRSRERARRERERKEELRGGGGDMAEPSEAGDAPPDDGPPGELGPDGPDGPEEKGRDRDRERRRSHRSERERRRDRDRDRDRDREHKRGERGSERGRDEARGGGGGQDNGLEGLGNDSRDMYMESEGGDGYLAPENGYLMEAAPE

**References**

Aquilina, J.A. (2008). The major toxin from the Australian Common Brown Snake is a hexamer with unusual gas-phase dissociation properties. Proteins.

Beausoleil, S.A., Villen, J., Gerber, S.A., Rush, J., and Gygi, S.P. (2006). A probability-based approach for high-throughput protein phosphorylation analysis and site localization. Nat Biotechnol *24*, 1285-1292.

Benesch, J.L.P., Aquilina, J.A., Ruotolo, B.T., Sobott, F., and Robinson, C.V. (2006). Tandem mass spectrometry reveals the quaternary organization of macromolecular assemblies. Chem Biol In press.

Dephoure, N., Zhou, C., Villen, J., Beausoleil, S.A., Bakalarski, C.E., Elledge, S.J., and Gygi, S.P. (2008). A quantitative atlas of mitotic phosphorylation. Proc Natl Acad Sci U S A *105*, 10762-10767.

Dieker, J., Cisterna, B., Monneaux, F., Decossas, M., van der Vlag, J., Biggiogera, M., and Muller, S. (2008). Apoptosis-linked changes in the phosphorylation status and subcellular localization of the spliceosomal autoantigen U1-70K. Cell Death Differ *15*, 793-804.

Dumortier, H., Klein Gunnewiek, J., Roussel, J.P., van Aarssen, Y., Briand, J.P., van Venrooij, W.J., and Muller, S. (1998). At least three linear regions but not the zinc-finger domain of U1C protein are exposed at the surface of the protein in solution and on the human spliceosomal U1 snRNP particle. Nucleic acids research *26*, 5486-5491.

Kuipers, B.J., and Gruppen, H. (2007). Prediction of molar extinction coefficients of proteins and peptides using UV absorption of the constituent amino acids at 214 nm to enable quantitative reverse phase high-performance liquid chromatography-mass spectrometry analysis. Journal of agricultural and food chemistry *55*, 5445-5451.

Muto, Y., Pomeranz Krummel, D., Oubridge, C., Hernandez, H., Robinson, C.V., Neuhaus, D., and Nagai, K. (2004). The structure and biochemical properties of the human spliceosomal protein U1C. J Mol Biol *341*, 185-198.

Olsen, J.V., Blagoev, B., Gnad, F., Macek, B., Kumar, C., Mortensen, P., and Mann, M. (2006). Global, in vivo, and site-specific phosphorylation dynamics in signaling networks. Cell *127*, 635-648.

Sobott, F., Benesch, J.L., Vierling, E., and Robinson, C.V. (2002). Subunit exchange of multimeric protein complexes. Real-time monitoring of subunit exchange between small heat shock proteins by using electrospray mass spectrometry. J Biol Chem *277*, 38921-38929.

Taverner, T., Hernandez, H., Sharon, M., Ruotolo, B.T., Matak-Vinkovic, D., Devos, D., Russell, R.B., and Robinson, C.V. (2008). Subunit architecture of intact protein complexes from mass spectrometry and homology modeling. Acc Chem Res *41*, 617-627.

van den Heuvel, R.H., van Duijn, E., Mazon, H., Synowsky, S.A., Lorenzen, K., Versluis, C., Brouns, S.J., Langridge, D., van der Oost, J., Hoyes, J., and Heck, A.J. (2006). Improving the Performance of a Quadrupole Time-of-Flight Instrument for Macromolecular Mass Spectrometry. Anal Chem *78*, 7473-7483.

Yu, L.R., Zhu, Z., Chan, K.C., Issaq, H.J., Dimitrov, D.S., and Veenstra, T.D. (2007). Improved titanium dioxide enrichment of phosphopeptides from HeLa cells and high confident phosphopeptide identification by cross-validation of MS/MS and MS/MS/MS spectra. Journal of proteome research *6*, 4150-4162.
