# Supplementary figures and images for: Isoforms of U1-70k Control Subunit Dynamics in the Human Spliceosomal U1 snRNP
Source: PLoS One. 2009 Sep 28;4(9):e7202. doi: 10.1371/journal.pone.0007202 (PMC2747018; doi:10.1371/journal.pone.0007202)

Figure S1

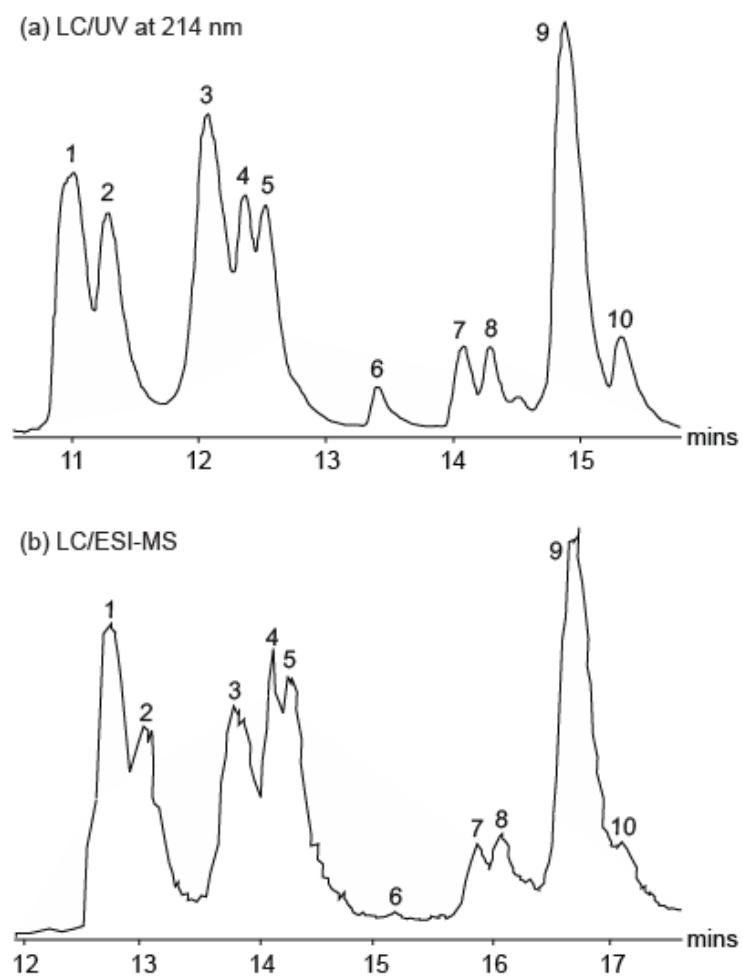

Supplement: Figure S1 — LC/UV (a) and LC/ESI-MS (b) chromatograms from U1 snRNP isolated from HeLa cells. The peak height ratio of Sm-B:Sm-B'in the UV (214 nm) trace is measured as 0.96. Using either only the number of amide bonds or calculated extinction co-efficients for amide bonds and all amino acid residues (Kuipers and Gruppen, 2007) gives a Sm-B:Sm-B' concentration ratio of 1.0. Protein identities were determined by LC/MALDI using on-plate tryptic digestion (see supplementary text). Protein identities: (1) Sm-D3, (2) Sm-B/B' fragment [13438 Da], (3) U1-C, (4) Sm-B', (5) Sm-B, (6) Sm-F, (7) Sm-G and U2 snRNP-A' [28284 Da], (8) U2 snRNP-B' [25529 Da], (9) U1-A, (10) Sm-E Peak 3 (Sm-B/B' fragment) was only observed in spectra from acidic solution conditions. The two U2 snRNP proteins were present as contaminants from purification. (0.24 MB PDF) [file pone.0007202.s002.pdf]

Figure S2

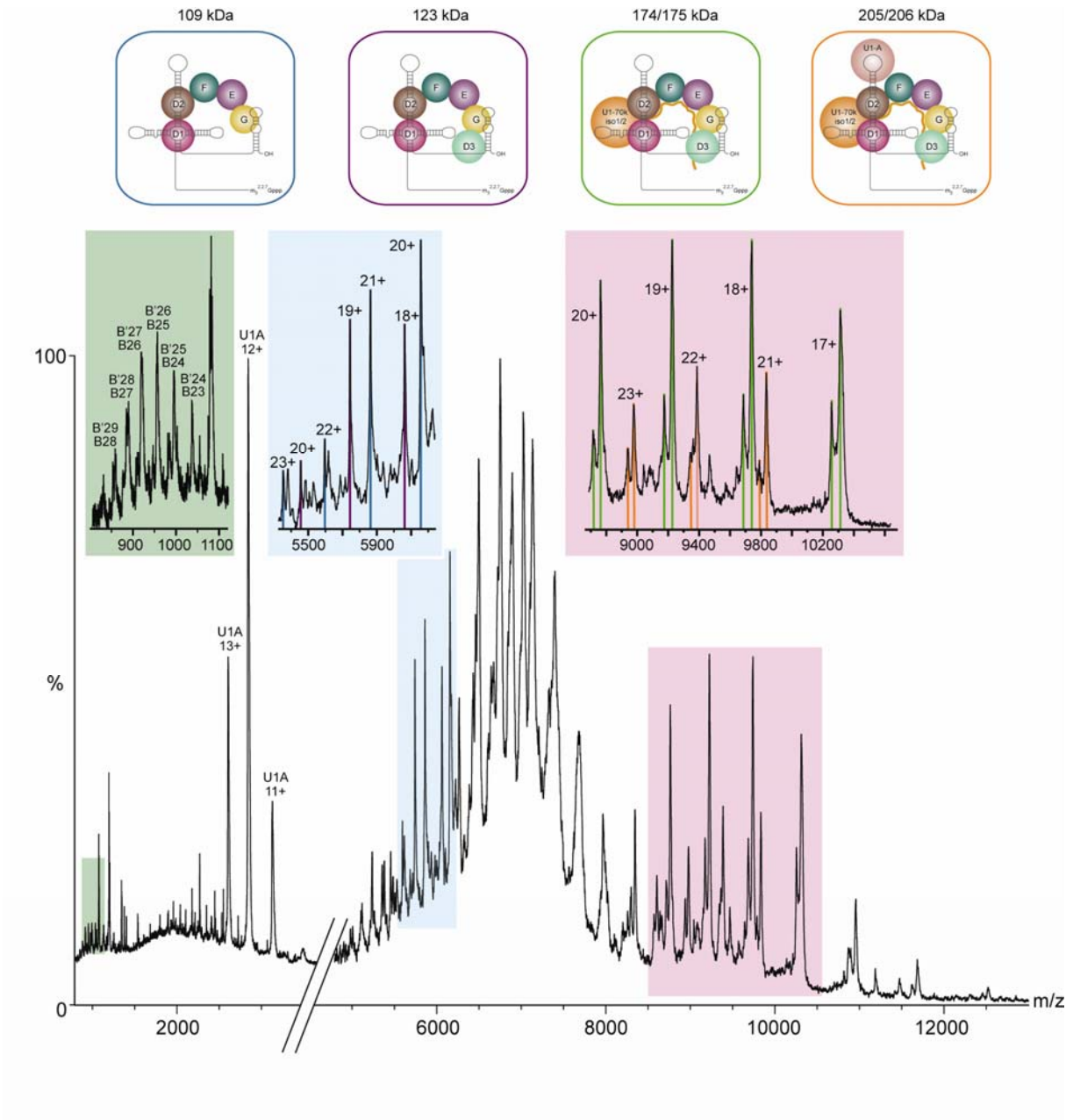

Supplement: Figure S2 — Electrospray mass spectra of the cellular U1 snRNP after buffer exchange to 150 mM ammonium acetate using centrifugal ultrafiltration followed by addition of butanol to give 128 mM ammonium acetate with 15% (v/v) butanol solution. Similar spectra were obtained from 150 mM ammonium acetate solution with no butanol present. The intact complex was not detected under these conditions. Predominant gas-phase dissociation of U1-C, Sm-B/B' and U1-A (pink inset) are observed together with two solution phase sub-complexes (blue inset) due to loss of all three U1-specific proteins together with Sm-B/B' and Sm-D3. At low m/z, Sm-B/B' is observed at unusually high charge states (green inset), consistent with an unfolded subunit. MS conditions: capillary: 1.3 kV, cone: 99 V, extractor: 100 V, collision cell voltage: 80 V, source readback: 3.4 mbar, analyser readback: 2.9×10^−4^ mbar, ToF readback: 1.1×10^−6^ mbar (0.33 MB PDF) [file pone.0007202.s003.pdf]

Figure S3

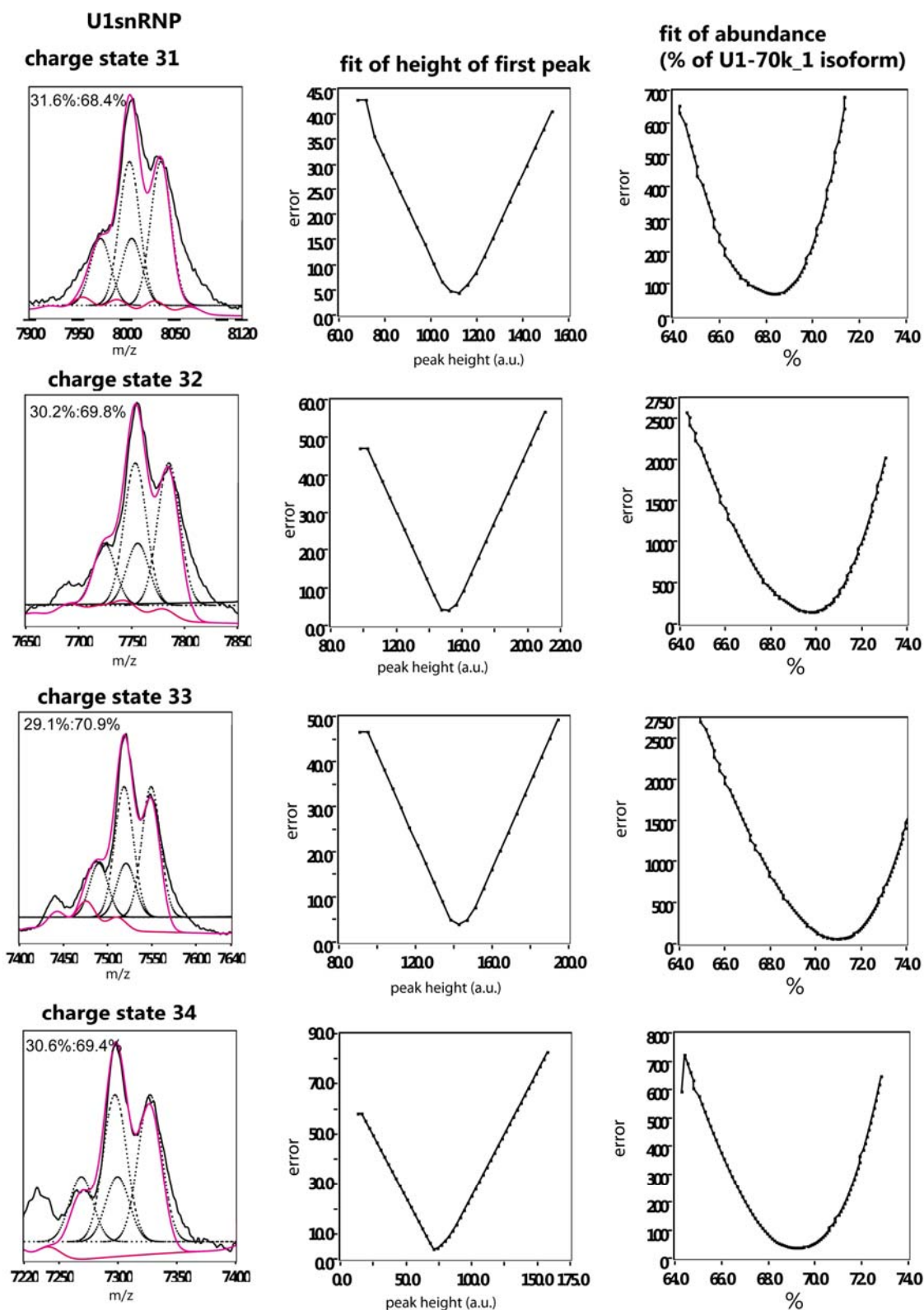

Supplement: Figure S3 — Fit of the intact U1snRNP complex. Fit of the U1snRNP complex with 4 Gaussians representing the different isoforms of subunit B/B' and U1-70k_1/_2. On the left the best fits for the different charge states of the complex are shown. The middle column shows the error function for the fit of the peak heights of the first peaks. The column on the right represents the fit for the abundance ratio of the U1-70k_1 isoform. The determined abundance is 30.2% : 69.8%. (0.37 MB PDF) [file pone.0007202.s004.pdf]

Figure S4

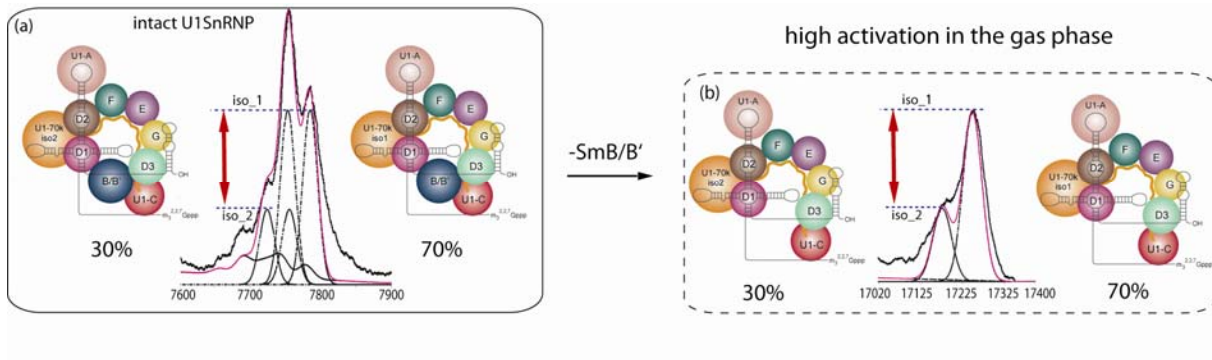

Supplement: Figure S4 — Higher energy activation: effect of product isoform ratio. Simulation of the peaks corresponding to the intact U1 snRNP containing different isoforms of SmB/B' and U1-70k isoforms 1 and 2. By monitoring the change in ratio of the peaks during dissociation of subunits we can assess potential interactions with various subunits. Under high activation conditions (collision cell voltage 160 V) the ratio of the U1-70k isoforms 1 and 2 however is indistinguishable from that in the intact complex. This in contrast to our results at lower activation conditions (collision cell 100 V) (figure 5 main text) where clear differences are observed in the ratio of the two isoforms after dissociation of Sm-B/B' and U1-C. (0.48 MB PDF) [file pone.0007202.s005.pdf]

Figure S5

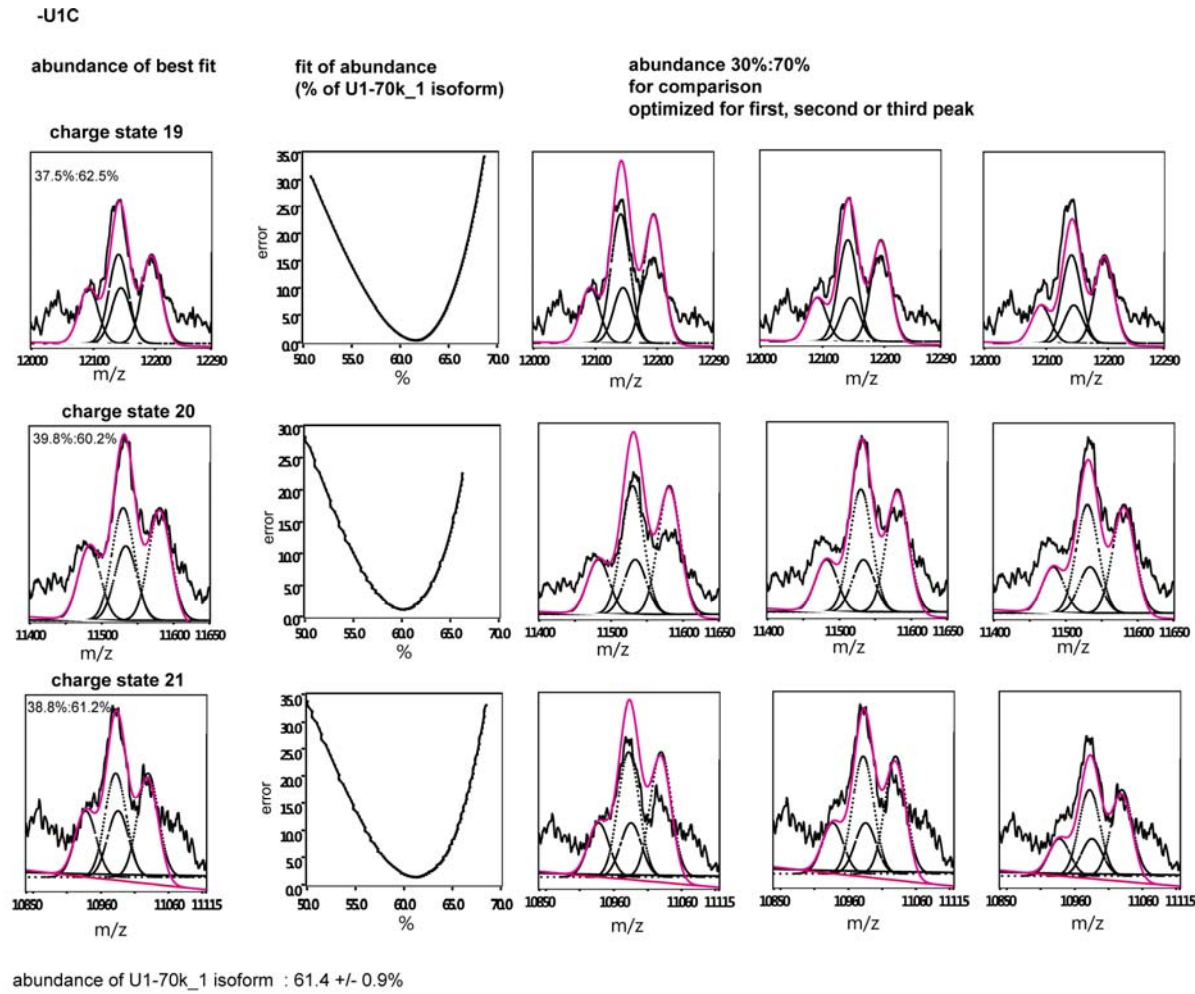

Supplement: Figure S5 — Fit of the -[U1-C] complex. Fit of the U1snRNP complex from which U1C has dissociated. The best fits obtained by minimizing the error of the fit and the spectra are shown (left). The error associated with the fitting is shown in the middle column. The abundance determined is 38.6% : 61.4% for U1-70k isoform 2:U1-70k isoform 1. For comparison three spectra on the right show Gaussian peaks representing the profile that would be obtained if there was no change in abundance with the peak height being optimized for the first, second or third peak from left to right respectively. (0.34 MB PDF) [file pone.0007202.s006.pdf]

Figure S7

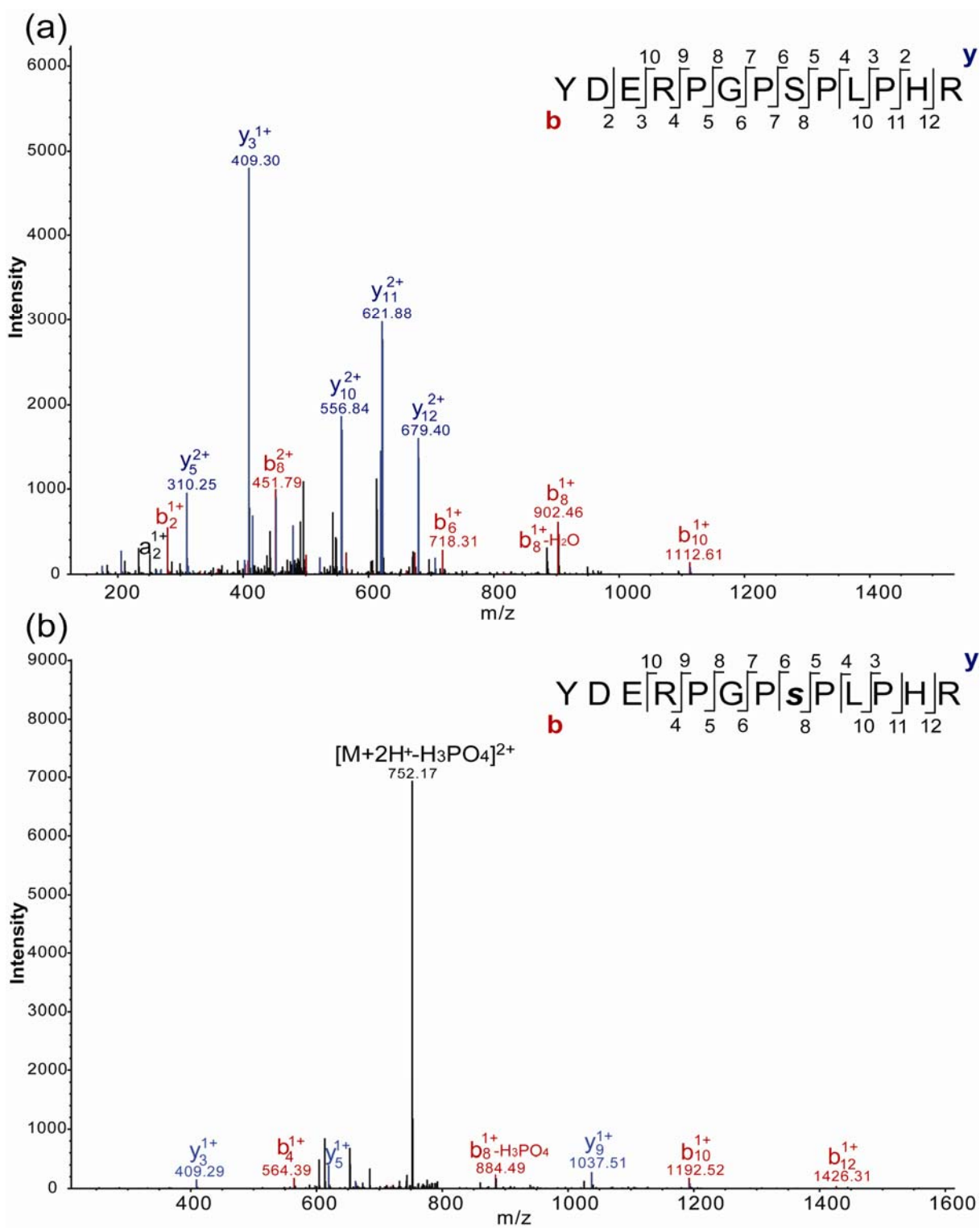

Supplement: Figure S7 — Phosphorylation of Ser226 in U1-70k isoform 1. Tandem MS spectra of the peptide Y_219 to R_231 which encompass the additional residues in U1-70k isoform 1 (a) and its phosphorylated from (b) recorded on the LTQ-Orbitrap after LC separation. Insets in (a) and (b) summarize series of b and y ions identified for these two peptides allowing confident assignment of their sequences and identification of a phosphorylation at Serine226. The tryptic digest was separated on a Ultimate 3000 HPLC system (Dionex) using a nanoC18 column with a 75 um i.d.. 0.1% formic acid was added to the mobile phase and the gradient was 0–45% acetonitrile in 30 minutes at a flow rate of 0.3 uL/min. Under these conditions the retention times for the phosphorylated and non-phosphorylated forms were 15.07 and 15.11 min.,respectively. Mass spectrometric analyses were performed using a hybrid LTQ orbitrap mass spectrometer (Thermo Fischer Scientific). Nano ESI was initiated by applying 1.85 kV to the picotip. The ion transfer capillary voltage and temperature were 35 V and 275°C respectively. The tube lens voltage was set to 110 V. External calibration was performed using the manufacturer's calibration mix. MS/MS was carried out using helium as collision gas and 6 scans were performed in the ion trap for the 6 most intense peaks per full scan at a normalized collisional energy of 35V and a maximum injection time of 100 ms. (0.31 MB PDF) [file pone.0007202.s008.pdf]
