# Supplementary material for: Isoforms of U1-70k Control Subunit Dynamics in the Human Spliceosomal U1 snRNP
Source: PLoS One. 2009 Sep 28;4(9):e7202. doi: 10.1371/journal.pone.0007202 (PMC2747018; doi:10.1371/journal.pone.0007202)
Supplement: Figure S6 — Fit of the -[B/B'] complex. Fit of the U1snRNP complex from which B/B' has dissociated. The best fits obtained by minimizing the error of the fit and the spectra are shown in the first column (left). The abundance determined is 23.3% : 76.7%. The error of the fits is shown in the middle column. For comparison the column on the right shows Gaussian peaks representing the distributions that would occur, if no change in the ratio of isoform abundance took place. (0.35 MB PDF) [file pone.0007202.s007.pdf]

Figure S6

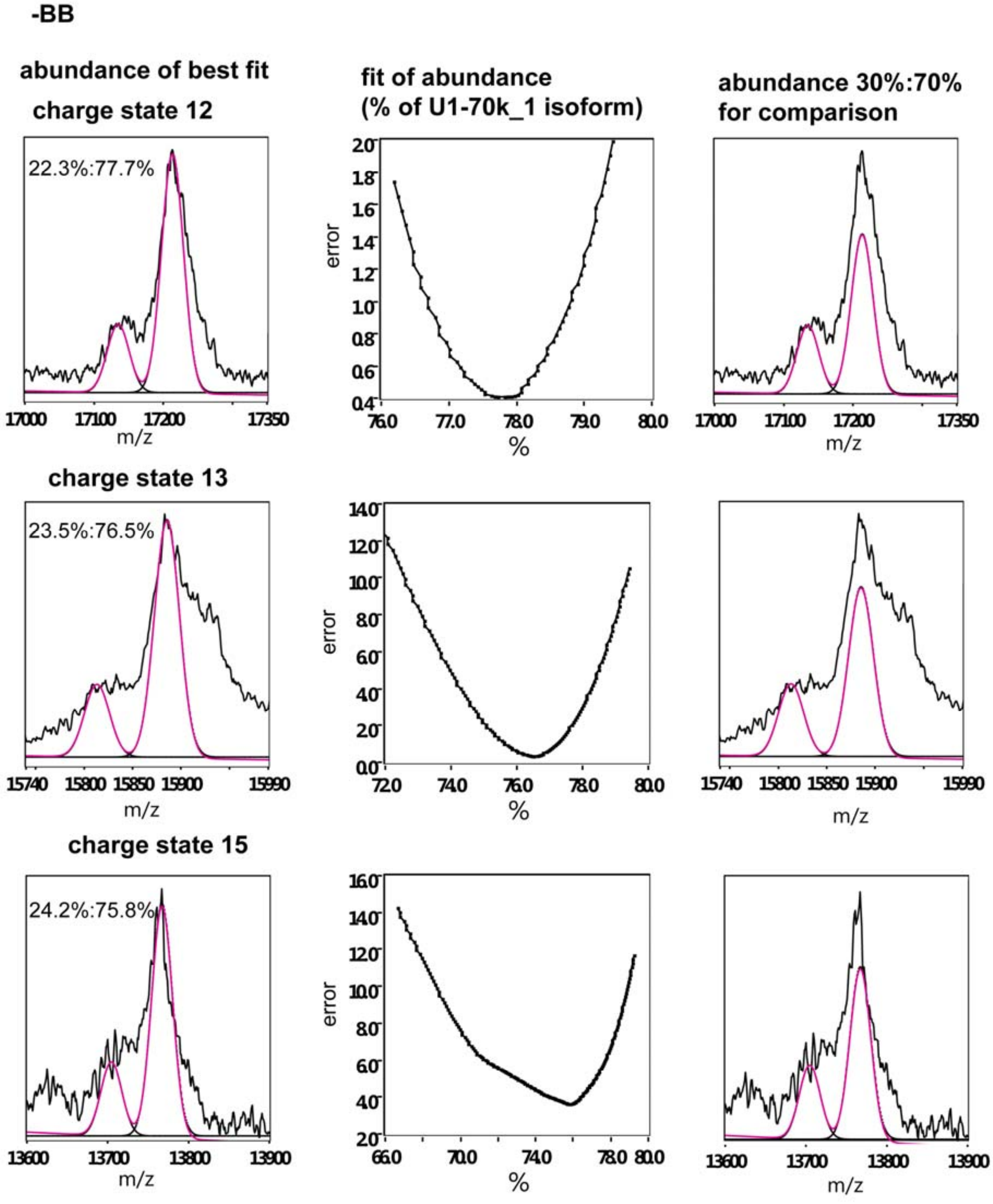

abundance of U1-70k_1 isoform : 76.7 +/- 1.0%
