# Supplementary material for: Isoforms of U1-70k Control Subunit Dynamics in the Human Spliceosomal U1 snRNP
Source: PLoS One. 2009 Sep 28;4(9):e7202. doi: 10.1371/journal.pone.0007202 (PMC2747018; doi:10.1371/journal.pone.0007202)
Supplement: Table S1 — Experimental and calculated masses of U1 snRNP proteins and RNA from HeLa cell complex (0.04 MB DOC) [file pone.0007202.s009.doc]

**Table S1**

| **Component** | **Swiss-Prot accession** | **Mass (Da)** | |
| --- | --- | --- | --- |
|  |  | **Experimental ± SD^a^** | **Sequence** |
| Sm-B | P14678-2 | 23749 ± 1.2 | 23656 |
| Sm-B’ | P14678-1 | 24761 ± 0.6 | 24610 |
| Sm-D_1_ | P62314 | n/d | 13282 |
| Sm-D_2_ | P62316 | n/d | 13527 |
| Sm-D_3_ | P62318 | 13966 ± 1.5 | 13916 |
| Sm-E | P62304 | 10743 ± 0.5 | 10804 |
| Sm-F | P62306 | 9636 ± 0.5 | 9725 |
| Sm-G | P62308| | 8407 ± 0.0 | 8496 |
| U1-A | P09012 | 31192 ± 0.7 | 31148 |
| U1-C | P09234 | 17373 ± 1.1 (17436)^b^ | 17394 |
| U1-70k isoform 1 | P08621-1 | n/d | 51557 |
| U1-70k isoform 2 | P08621-2 | n/d | 50618 |
| U1snRNA | - | 53250 ± 22 | 53271^c^ |

^a^ average experimental mass and standard deviation (SD) from at least three measurements from denaturing solution conditions

^b^ experimental mass + 63 Da to include one bound Zn^2+^ under native conditions (Dumortier et al., 1998; Muto et al., 2004) for the reported mass from Masslynx (additional reported mass = Zn mass - 2H mass)

^c^ Branlant et al. 1980. Sequence mass includes trimethylguanosine cap and several modified nucleotides.

n/d not detected
