# Supplementary material for: Isoforms of U1-70k Control Subunit Dynamics in the Human Spliceosomal U1 snRNP
Source: PLoS One. 2009 Sep 28;4(9):e7202. doi: 10.1371/journal.pone.0007202 (PMC2747018; doi:10.1371/journal.pone.0007202)
Supplement: Table S2 — Calculated masses of intact U1 snRNP and sub-complexes. (0.03 MB DOC) [file pone.0007202.s010.doc]

**Table S2**

|  | **Intact mass (Da)** | | **[-U1C] (Da)** | | **[-B/B'-U1C]**  **(Da)** | **[-B/B'-U1C-U1A]**  **(Da)** |
| --- | --- | --- | --- | --- | --- | --- |
| **U1-70k** | **Sm-B** | **Sm-B'** | **Sm-B** | **Sm-B'** |
| **isoform 1** | 246745 | 247757 | 229309 | 230321 | 205560 | 174368 |
| **isoform 2** | 245806 | 246818 | 228370 | 229382 | 204621 | 173429 |
