# Supplementary material for: Isoforms of U1-70k Control Subunit Dynamics in the Human Spliceosomal U1 snRNP
Source: PLoS One. 2009 Sep 28;4(9):e7202. doi: 10.1371/journal.pone.0007202 (PMC2747018; doi:10.1371/journal.pone.0007202)
Supplement: Table S3 — Masses of complexes, subcomplexes and single proteins. (0.14 MB DOC) [file pone.0007202.s011.doc]

**Table S3**

**Spectrum 1**

Sample: U1/1

Solution: 200 mM ammonium acetate

MS (Q2): 1.5/200/0/100 7.1x10^-3^, 1.x10^-6^ mbar

| **Experimental mass ± SD^1^** | **Identity^2^** | **Complex type** |
| --- | --- | --- |
| 216954 ± 117 mj | [int: SmB: U170k_1] – U1A  [int: SmB’: U170k_2] – U1A | sp |
| 217899 ± 124 mn | [int: SmB’: U170k_1] - U1A | sp |
| 247167 ± 56 mn | [int: SmB: U170k_2] | sp |
| 248150 ± 19 mj | [int: SmB: U170k_1]  [int: SmB’: U170k_2] | sp |
| 249052 ± 34 mn | [int: SmB’: U170k_1] | sp |
| 230626 ± 64 | [int: SmB: U170k_1] – U1C  [int: SmB’: U170k_2] – U1C | sp |
| 206480 ± 35 | [int: U170k_1] – U1C | 2^nd^ CID |
| 223978 ± 17 | [int: U170k_1] | CID |
| 230581 ± 77 | [int: SmB: U170k_1] – U1C  [int: SmB’: U170k_2] – U1C | CID |
| 236443 ± 82 mn | [int: SmB: U170k_2] - SmE | CID |
| 237248 ± 34 mj | [int: SmB: U170k_1] - SmE  [int: SmB’: U170k_2] - SmE | CID |
| 238327 ± 63 mn | [int: SmB’: U170k_1] - SmE | CID |
|  |  |  |
| 8406 ± 0.3 | SmG |  |
| 9635 ± 0.3 | SmF |  |
| 10742 ± 0.2 | SmE |  |
| 10773 ± 0.3 | SmE+31 |  |
| 13966 ± 1.5 | SmD3 |  |
| 13980 ± 0.8 | SmD3+14 |  |
| 17373 ± 0.9 | U1C |  |
| 17400 ± 2.7 | U1C+27 |  |
| 17428 ± 2.5 | U1C+55 |  |
| 17448 ± 4.1 | U1C+75 |  |
| 23747 ± 1.1 | SmB |  |
| 24758 ± 2.0 | SmB’ |  |
| 31189 ± 0.3 | U1A |  |
| 31230 ± 0.9 | U1A+41 |  |

**Spectrum 2**

Sample: U1/1

Solution: 200 mM ammonium acetate

MS (Q2): 1.5/200/0/130 7.1x10^-3^, 1.3x10^-6^ mbar

| **Experimental mass ± SD^1^** | **Identity^2^** | **Complex type** |
| --- | --- | --- |
| 216963 ± 105 mj | [int: SmB: U170k_1] – U1A  [int: SmB’: U170k_2] – U1A | sp |
| 217888 ± 101 mn | [int: SmB’: U170k_1] - U1A | sp |
| 230662 ± 25 | [int: SmB: U170k_1] – U1C  [int: SmB’: U170k_2] – U1C | sp |
| 247118 ± 57 mn | [int: SmB: U170k_2] | sp |
| 248147 ± 59 mj | [int: SmB: U170k_1]  [int: SmB’: U170k_2] | sp |
| 249088 ± 51 mn | [int: SmB’: U170k_1] | sp |
| 206301 ± 85 | [int: U170k_1] – U1C | 2^nd^ CID |
| 224175 ± 39 | [int: U170k_1] | CID |
| 229574 ± 33 mn | [int: SmB: U170k_2] – U1C | CID |
| 230545 ± 87 mj | [int: SmB: U170k_1] – U1C  [int: SmB’: U170k_2] – U1C | CID |
| 231513 ± 64 mn | [int: SmB: U170k_2] – U1C | CID |
| 236410 ± 65 mn | [int: SmB: U170k_2] - SmE | CID |
| 237220 ± 65 mj | [int: SmB: U170k_1] - SmE  [int: SmB’: U170k_2] - SmE | CID |
| 238260 ± 48 mn | [int: SmB’: U170k_1] - SmE | CID |
|  |  |  |
| 8406 ± 1.1 | SmG |  |
| 9635 ± 0.4 | SmF |  |
| 10742 ± 0.4 | SmE |  |
| 10774 ± 0.2 | SmE+32 |  |
| 13967 ± 1.4 | SmD3 |  |
| 13980 ± 0.9 | SmD3+13 |  |
| 17376 ± 4.1 | U1C |  |
| 17400 ± 3.1 | U1C+24 |  |
| 17426 ± 1.3 | U1C+50 |  |
| 17452 ± 3.5 | U1C+76 |  |
| 17477 ± 1.4 | U1C+101 |  |
| 23748 ± 0.4 | SmB |  |
| 24758 ± 1.3 | SmB’ |  |
| 31189 ± 0.9 | U1A |  |
| 31231 ± 1.0 | U1A+42 |  |

**Spectrum 3**

Sample: U1/1

Solution: 200 mM ammonium acetate

MS (Q2): 1.5/200/0/160 7.1x10^-3^, 1.3x10^-6^ mbar

| **Experimental mass ± SD^1^** | **Identity^2^** | **Complex type** |
| --- | --- | --- |
| 213319 ± 27 | [int: U170k_1] - SmE | CID |
| 219817 ± 34 eq | [int: SmB: U170k_1] – U1C -SmE  [int: SmB’: U170k_2] – U1C - SmE | CID |
| 220867 ± 57 eq | [int: SmB: U170k_2] – U1C – SmE­ | CID |
| 223308 ± 58 mn | [int: U170k_2] | CID |
| 224199 ± 35 mj | [int: U170k_1] | CID |
| 230621 ± 94 mj | [int: SmB: U170k_1] – U1C  [int: SmB’: U170k_2] – U1C | CID |
| 231608 ± 101 mn | [int: SmB: U170k_2] – U1C | CID |
|  |  |  |
| 8407 ± 1.2 | SmG |  |
| 9636 ± 0.5 | SmF |  |
| 10742 ± 0.4 | SmE |  |
| 10774 ± 0.2 | SmE+32 |  |
| 17372 ± 4.1 | U1C |  |
| 17400 ± 2.9 | U1C+28 |  |
| 17421 ± 4.5 | U1C+49 |  |
| 23749 ± 0.3 | SmB |  |
| 24759 ± 1.4 | SmB’ |  |
| 31190 ± 1.4 | U1A |  |
| 31231 ± 1.0 | U1A+41 |  |

**Spectrum 4**

Sample: U1/3

Solution: 200 mM ammonium acetate, pH 7.9

MS (Q2): 1.5/200/100/50 1.0x10^-2^, 2.9x10^-4^, 1.4e-6 mbar

| **Experimental mass ± SD^1^** | **Identity^2^** | **Complex type** |
| --- | --- | --- |
| 175359 ± 32 | [int: U170k_1] – U1C – U1A | sp |
| 206648 ± 39 | [int: U170k_1] – U1C | sp |
| 230404 ± 54 | [int: SmB: U170k_1] – U1C  [int: SmB’: U170k_2] – U1C | sp |
| 154925 ± 23 | [int: U170k_1] – U1C – U1A – SmE - SmF | 2^nd^ CID |
| 164581 ± 41 | [int: U170k_1] – U1C – U1A - SmE | CID |
| 164504 ± 101 | [int: U170k_1] – U1C – U1A - SmE | 2^nd^ CID |
| 174344 ± 21 mn | [int: U170k_2] – U1C – U1A | CID |
| 175294 ± 25 mj | [int: U170k_1] – U1C – U1A | CID |
| 206487 ± 32 | [int: U170k_1] – U1C | CID |
|  |  |  |
| 8406 ± 1.2 | SmG |  |
| 9635 ± 0.4 | SmF |  |
| 10741 ± 0.3 | SmE |  |
| 10773 ± 0.2 | SmE+32 |  |
| 13965 ± 0.7 | SmD3 |  |
| 13979 ± 0.2 | SmD3+14 |  |
| 31188 ± 0.4 | U1A |  |

**Spectrum 5**

Sample: U1 60T

Solution: 128 mM ammonium acetate, 15% (v/v) butanol

| **Experimental mass ± SD^1^** | **Identity^2^** | **Complex type** |
| --- | --- | --- |
| 109103 ± 6 | [int] - U1C – U1A – SmD3 | sp |
| 123108 ± 28 | [int] - U1C – U1A | sp |
| 175483 ± 78 | [int: U170k_1] – U1C – U1A | ? |
| 206802 ± 75 | [int: U170k_1] – U1C | ? |
| 123041 ± 18 | [int] - U1C – U1A | CID |
| 174327 ± 36 mn | [int: U170k_2] – U1C – U1A | CID |
| 175279 ± 30 mj | [int: U170k_1] – U1C – U1A | CID |
| 205568 ± 40 | [int: U170k_2] – U1C | CID |
| 206498 ± 43 | [int: U170k_1] – U1C |  |
|  |  |  |
| 8406 ± 1.2 | SmG |  |
| 9636 ± 0.9 | SmF |  |
| 10742 ± 0.8 | SmE |  |
| 10774 ± 0.7 | SmE+32 |  |
| 23742 ± 5.5 | SmB |  |
| 31185 ± 1.7 | U1A |  |

**Spectrum 6**

Sample: U1 60T

Solution: 150 mM ammonium acetate, pH 7.7

MS (Q2B): 1.3/99/100/80 3.4, 1.0x10^-4^, 1.0x10^-6^ mbar

| **Experimental mass ± SD^1^** | **Identity^2^** | **Complex type** |
| --- | --- | --- |
| 109075 ± 32 | [int] - U1C – U1A – SmD3 | sp |
| 123058 ± 16 | [int] - U1C – U1A | sp |
| 160392 ± 82 | [int: U170k_2] – U1C – U1A – SmD3 | sp |
| 161310 ± 78 | [int: U170k_1] – U1C – U1A – SmD3 | sp |
| 174331 ± 44 mn | [int: U170k_2] – U1C – U1A | sp |
| 175306 ± 26 mj | [int: U170k_1] – U1C – U1A | sp |
| 205822 ± 63 eq | [int: U170k_2] – U1C | sp |
| 206619 ± 52 wq | [int: U170k_1] – U1C | sp |
| 150508 ± 22 | [int: U170k_1] – U1C – U1A – SmD3 - SmE | CID |
| 164514 ± 15 | [int: U170k_1] – U1C – U1A - SmE | CID |
| 174249 ± 29 mn | [int: U170k_2] – U1C – U1A | CID |
| 175228 ± 12 mj | [int: U170k_1] – U1C – U1A | CID |
| 206442 ± 25 | [int: U170k_1] – U1C |  |
|  |  |  |
| 8406 ± 1.2 | SmG |  |
| 9634 ± 0.7 | SmF |  |
| 10741 ± 0.2 | SmE |  |
| 10773 ± 0.2 | SmE+32 |  |
| 13965 ± 1.2 | SmD3 |  |
| 13979 ± 1.8 | SmD3+14 |  |
| 23746 ± 2.7 | SmB |  |
| 31185 ± 1.7 | U1A |  |

^1^average experimental mass ± SD calculated from at least three charge states measured at the peak centre

^2^ [int] = [RNA1: U1A: U1C: SmD1: SmD2: SmD3: SmE: SmF: SmG]

U170k_1 and U170_2 refer to U1-70k isoforms 1 and 2 respectively

mj: major species of pair

mn: minor species of pair

eq: approximately equal intensity

sp: solution phase complex

CID: collision-induced dissociation product

MS parameters are reported as capillary/cone/extractor/collision cell voltages followed by pressure readbacks.

'Q2' and 'Q2B' instruments are both modified QToF2 mass spectrometers.
