# Supplementary material for: Isoforms of U1-70k Control Subunit Dynamics in the Human Spliceosomal U1 snRNP
Source: PLoS One. 2009 Sep 28;4(9):e7202. doi: 10.1371/journal.pone.0007202 (PMC2747018; doi:10.1371/journal.pone.0007202)
Supplement: Table S4 — Reported phosphorylation sites in U1-70k from HeLa cells. (0.05 MB DOC) [file pone.0007202.s012.doc]

**Table S4**

| **Residue** | **Isoform** | **Reference^1^** |
| --- | --- | --- |
| S140 | 1 | (Dieker et al., 2008) |
| S140 | 2 |
| S226 | 1 | (Dephoure et al., 2008; Yu et al., 2007) |
| S266 | 1 | (Olsen et al., 2006) |
| S257 | 2 |
| S268 | 1 | (Olsen et al., 2006) |
| S259 | 2 |
| S320 | 1 | (Dephoure et al., 2008; Olsen et al., 2006) |
| S311 | 2 |
| S410 | 1 | (Beausoleil et al., 2006; Dephoure et al., 2008; Olsen et al., 2006) |
| S401 | 2 |

^1^ From http://www.phosphosite.org and http://www.uniprot.org
