# Supplementary material for: Isoforms of U1-70k Control Subunit Dynamics in the Human Spliceosomal U1 snRNP
Source: PLoS One. 2009 Sep 28;4(9):e7202. doi: 10.1371/journal.pone.0007202 (PMC2747018; doi:10.1371/journal.pone.0007202)
Supplement: Table S5 — Parameters used to model experimental spectra. The mass shifts were added to the theoretical masses of the (sub)complexes prior to modeling. This enabled matching of the mean of each peak Gaussian with the corresponding peak top in the experimental spectrum. These mass shifts are due to buffer, water and salt molecules that adhere to the protein complex and were found to be of a lower value for the CID complexes than for the solution phase complexes. Varying resolution of the peaks as well as different adducts lead to broad peaks in the spectra which is reflected in their different full width at half maximum (FWHM) values. (0.03 MB DOC) [file pone.0007202.s013.doc]

**Table S5**

|  | spectrum1 | spectrum 2 | spectrum 3 | spectrum 4 | spectrum 5 | spectrum 6 |
| --- | --- | --- | --- | --- | --- | --- |
| mass shift (Da) solution phase  complexes | 1370 | 1235 | 1235 | 1000 | 1000 | 900 |
| mass shift (Da)  CID complexes | 1100 | 1050 | 1235 | 850 | 900 | 800 |
| FWHM (m/z) | 25-30 | 25-37 | 37-53 | 17 | 21 | 21-27 |
