# Supplementary material for: Isoforms of U1-70k Control Subunit Dynamics in the Human Spliceosomal U1 snRNP
Source: PLoS One. 2009 Sep 28;4(9):e7202. doi: 10.1371/journal.pone.0007202 (PMC2747018; doi:10.1371/journal.pone.0007202)
Supplement: Table S6 — Possible compositions of 109 kDa and 123 kDa subcomplexes (0.04 MB DOC) [file pone.0007202.s014.doc]

**Table S6**

**(a) 109 kDa**

| **Peak centre experimental mass: 109089 ± 20 Da** | | |
| --- | --- | --- |
| **Composition** | **Mass (Da)^a^** | **ΔM^b^** |
| G:F:D1:B':RNA | 109336 | -247 |
| F:E:D2:B':U170k_2 | 109285 | -196 |
| F:E:D2:B:U170k_1 | 109212 | -123 |
| B':U1A:RNA | 109203 | -114 |
| F:E:D1:B':U170k_2 | 109040 | 49 |
| G:E:D2:B':U170k_1 | 108995 | 94 |
| D2:U1C:B':RNA | 108974 | 115 |
| F:E:D1:B:U170k_1 | 108967 | 122 |
| **F:U1C:U1A:U170k_2** | **108882** | **207** |
| **G:F:E:D1:D2:RNA** | **108845** | **244** |
| F:D1:D2:U1C:B:U1A | 108822 | 267 |

**(b) 123 kDa**

| **Peak centre experimental mass: 123069 ± 35 Da** | | |
| --- | --- | --- |
| **Composition** | **Mass (Da)^a^** | **ΔM^b^** |
| G:F:D1:D3:B':RNA | 123302 | -233 |
| F:E:D2:D3:B':U170k_2 | 123251 | -182 |
| F:E:D2:D3:B:U170k_1 | 123178 | -109 |
| D3:B':U1A:RNA | 123169 | -100 |
| F:E:D1:D3:B':U170k_2 | 123006 | 63 |
| G:E:D2:D3:B':U170k_1 | 122961 | 108 |
| D2:D3:U1C:B':RNA | 122940 | 129 |
| F:E:D1:D3:B:U170k_1 | 122933 | 136 |
| **F:D3:U1C:U1A:U170k_2** | **122848** | **221** |
| **G:F:E:D1:D2:D3:RNA** | **122811** | **258** |
| F:D1:D2:D3:U1C:B:U1A | 122788 | 281 |

Hit lists generated using SUMMIT with the following limits: ± 0.25 % mass tolerance, 123 kDa must contain Sm-D3, 109 kDa must not contain Sm-D3, single copy only of possible proteins. Hits in bold include the additional limit that they do not contain Sm-B/B' as the charge states for 123 kDa and 109 kDa are single peaks.

^a^ calculated mass from individual masses in table S3

^b^ ΔM = Experimental mass – Calculated mass (Da)
