# Supplementary material for: Isoforms of U1-70k Control Subunit Dynamics in the Human Spliceosomal U1 snRNP
Source: PLoS One. 2009 Sep 28;4(9):e7202. doi: 10.1371/journal.pone.0007202 (PMC2747018; doi:10.1371/journal.pone.0007202)
Supplement: Table S7 — Dataset of masses used for input in SUMMIT. (0.03 MB DOC) [file pone.0007202.s015.doc]

**Table S7**

| **Component** | **Mass (Da)** |
| --- | --- |
| Sm-B | 23749 |
| Sm-B’ | 24761 |
| Sm-D1 | *13282* |
| Sm-D2 | *13527* |
| Sm-D3 | 13966 |
| Sm-E | 10743 |
| Sm-F | 9636 |
| Sm-G | 8407 |
| U1-A | 31192 |
| U1-C | 17436 |
| U1-70k isoform 1 | *51557* |
| U1-70k isoform 2 | *50618* |
| U1snRNA | 53250 |

Entries in italics are sequence masses (see table S1)
