# Supplementary material for: Isoforms of U1-70k Control Subunit Dynamics in the Human Spliceosomal U1 snRNP
Source: PLoS One. 2009 Sep 28;4(9):e7202. doi: 10.1371/journal.pone.0007202 (PMC2747018; doi:10.1371/journal.pone.0007202)
Supplement: Table S8 — Average mass differences and protein identities. (0.04 MB DOC) [file pone.0007202.s016.doc]

**Table S8**

| **ΔM^1^** | **ΔM from single protein^2^** | **Protein / subcomplex^3^** | **Note^4^** |
| --- | --- | --- | --- |
| 10802 ± 97 | 103 | **E** |  |
| 10773 ± 45 | 30 | **E** |  |
| 17557 ± 55 | 119 | **U1C** |  |
| 17686 ± 267 | 248 | - | 1.40% = **U1-C** |
| 31183 ± 21 | -9 | D3/U1C or G/F/D1 or D2/U1C or **U1A** |  |
| 31218 | 26 | G/F/D1 or D3/U1C or D2/U1C or **U1A** |  |
| 31407 ± 108 | 215 | D3/U1C or G/F/D2 or G/F/D1 or **U1A** |  |
| 24072 ± 141 | 323 | E/D2 or E/D1 | 1.34% = **B** or E/D1 or E/D2 |
| 24994 ± 114 | 233 | **B'** |  |
| 24002 ± 212 | 253 | E/D1 | 1.05% = **B** or E/D1 |
| 25212 | 451 | - | 1.79% = **B'** |
| 23875 ± 25 | 126 | **B** or E/D1 |  |
| 10880 | 137 | - | 1.26% = **E** |
| 10786 ± 7 | 43 | **E** |  |
| 10802 | 59 | **E** |  |
| 13996 | 30 | **D3** |  |
| 14006 | 40 | **D3** |  |
| 9656 | 20 | **F** |  |
| 14005 ± 30 | 39 | **D3** |  |

^1^ Average mass differences from figure 4 (main text)

2 [average mass difference - single protein mass], see table S3

^3^ Possible compositions for a given mass difference determined using SUMMIT with a ± 1% mass tolerance

^4^ Mass tolerance required to give a single protein hit

Bold type indicates single protein hit
