# Supplementary material for: Isoforms of U1-70k Control Subunit Dynamics in the Human Spliceosomal U1 snRNP
Source: PLoS One. 2009 Sep 28;4(9):e7202. doi: 10.1371/journal.pone.0007202 (PMC2747018; doi:10.1371/journal.pone.0007202)
Supplement: Table S9 — Experimental masses of U1 snRNP proteins and RNA from the recombinant complex. (0.03 MB DOC) [file pone.0007202.s017.doc]

**Table S9**

| **Component** | **Mass (Da)** |
| --- | --- |
| Sm-B (1-174) | 17848 |
| Sm-D1(full, 1-119) | 13282 |
| Sm-D2 (full, 1-118) | 13396 |
| Sm-D3 (full, 1-126) | 13961 |
| Sm-E (full, 1-92) | 10672 |
| Sm-F (1-86) | 9594 |
| Sm-G (His tag, 1-76) | 9448 |
| U1-A (full, 1-282) | 32140 |
| U1-70k (1-216) | 25322 |
| U1snRNA (1-165) | 52862 |
